# Supplementary material for: Ultra High-plex Spatial Proteogenomic Investigation of Giant Cell Glioblastoma Multiforme Immune Infiltrates Reveals Distinct Protein and RNA Expression Profiles
Source: Cancer Res Commun. 2023 May 3;3(5):763–79. doi: 10.1158/2767-9764.CRC-22-0396 (PMC10155752; doi:10.1158/2767-9764.CRC-22-0396)
Supplement: Supplementary Tables S1-S6 — Supplementary Table S1: A list of materials and reagents that are recommended for the GeoMx DSP assays that are not supplied by NanoString. Supplementary Table S2. A list of NanoString supplied materials and reagents. Supplementary Table S3: A list of Human protein targets by module. Supplementary Table S4: A list of Mouse protein targets by module. Supplementary Table S5: The 11-cell pellet array (CPA) used in assay development. Supplementary Table S6: The 45-cell pellet array (CPA) used in assay development. [file crc-22-0396-s01.pdf]

# Supplementary Tables

**Supplementary Table S1:** A list of materials and reagents that are recommended for the GeoMx DSP assays that are not supplied by NanoString.

| Reagent/Material | Source/Part Number |
|---|---|
| 10% neutral buffered formalin (NBF) | EMS Diasum, 15740-04 |
| 100% deionized formamide | VWR, VWRV0606 |
| 100% ethanol (EtOH): ACS grade or better | Various |
| 10X citrate buffer pH 6 | Sigma-Aldrich, C9999-1000ML |
| 10X phosphate buffered saline pH 7.4 (PBS) | Invitrogen, 00652158 |
| 10X TBS with tween 20 (TBS-T) | Cell Signaling Technologies, 9997S |
| 10X tris buffered saline (TBS) | Cell Signaling Technologies, 12498S |
| 16% paraformaldehyde (PFA) | 16% stock, 28908 |
| 1X phosphate buffered saline pH 7.4 (PBS) | ThermoFisher,10010031 |
| 20X SSC (DNase, RNase free) | Sigma-Aldrich, S6639 |
| BOND Dewax Solution, 1 L | Leica Biosystems, AR9222 |
| BOND Epitope Retrieval 1, 1 L | Leica Biosystems, AR9961 |
| BOND Epitope Retrieval 2, 1 L | Leica Biosystems, AR9640 |
| BOND Wash Solution 10X Concentrate, 1 L | Leica Biosystems, AR9590 |
| CitriSolv | Fisher Scientific, 04-355-121 |
| DEPC-treated water | ThermoFisher, AM9922 |
| Glycine | Sigma-Aldrich, G7126 |
| Proteinase K | Ambion, AM2546 |
| Tris base | Sigma-Aldrich, 10708976001 |
| HybriSlip hybridization covers (22 mm x 40 mm x 0.25 mm) | Grace Bio-Labs, 714022 |
| RNase Away | Thermofisher, 7003PK |
| Dextran sulfate | Sigma, 67578 |
| BSA | VWR, 97061-420 |
| Deoxyribonucleic acid, single stranded from salmon testes (denatured by heating at 95°C for 10 min prior to use) | Sigma, D7656 |
| Agencourt AMPure XP | Beckman Coulter, A63880 |
| Elution buffer (Tris-HCl 10 mM with 0.05% Tween-20, pH 8.0) * | Teknova, T1485 |
| Bioanalyzer DNA High Sensitivity Kit | Agilent, 5067-4626 |
| Quibit dsDNA HS Assay Kit | Q32854 |
| GFAP antibody (GA-5) Alexa Fluor® 647 | Novus Biologicals, NPB2-33184AF647, RRID:AB_2935766 |
| nCounter Master Kit (Maxor FLEX Systems) Reagents and Cartridges | NAA-AKIT-012 |
| BOND Research Detection System (includes 6x 30 mL Open Containers) | Leica Biosystems, DS9455 |
| BOND Titration Kit (includes 50 inserts) | Leica Biosystems, OPT9049 |
| BOND Universal Covertiles | Leica Biosystems, S21.2001 |
| BOND Open Containers 30 mL | Leica Biosystems, OP309700 |

**Supplementary Table S2.** A list of NanoString supplied materials and reagents.

| Reagent/Material | Source/Item |
|---|---|
| GeoMx Solid Tumor TME Morphology Kit Human Protein Compatible | Nanostring Technologies, GMX-PRO-MORP-HHST-12 |
| GeoMx Solid Tumor TME Morphology Kit Mouse RNA FFPE Compatible | Nanostring Technologies, GMX-RNA-MORPH-MST-FFPE12 |
| GeoMx Immune Cell Profiling Panel Human Protein Core for nCounter | Nanostring Technologies, GMX-PROCO-NCT-HICP-12 |
| GeoMx Immune Activation Status Panel Human Protein Module for nCounter | Nanostring Technologies, GMX-PROMOD-NCT-HICT(AS)-12 |
| GeoMx Immune Cell Typing Panel Human Protein Module for nCounter | Nanostring Technologies, GMX-PROMOD-NCT-HICT-12 |
| GeoMx IO Drug Target Panel Human Protein Module for nCounter | Nanostring Technologies, GMX-PROMOD-NCT-HIODT-12 |
| GeoMx Human Protein Core for NGS | Nanostring Technologies, GMX-PROCO-NGS-HCORE-12 |
| GeoMx Immune Cell Typing Panel Human Protein Module for NGS | Nanostring Technologies, GMX-PROMOD-NGS-HICT-12 |
| GeoMx IO Drug Target Panel Human Protein Module for NGS | Nanostring Technologies, GMX-PROMOD-NGS-HIODT-12 |
| GeoMx Immune Activation Status Panel Human Protein Module for NGS | Nanostring Technologies, GMX-PROMOD-NGS-HIAS-12 |
| GeoMx Pan-Tumor Panel Human Protein Module for NGS | Nanostring Technologies, GMX-PROMOD-NGS-HPT-12 |
| GeoMx Myeloid Panel Human Protein Module for NGS | Nanostring Technologies, GMX-PROMOD-NGS-HMY-12 |
| GeoMx MAPK Signaling Panel Human Protein Module for NGS | Nanostring Technologies, GMX-PROMOD-NGS-HMAPK-12 |
| GeoMx PI3K/AKT Signaling Panel Human Protein Module for NGS | Nanostring Technologies, GMX-PROMOD-NGS-HPI3K-12 |
| GeoMx Neural Cell Typing Panel Human Protein Module for NGS | Nanostring Technologies, GMX-PROMOD-NGS-HNCT-12 |
| GeoMx Alzheimer's Pathology Panel Human Protein Module for NGS | Nanostring Technologies, GMX-PROMOD-NGS-HADP-12 |
| GeoMx Parkinson's Pathology Panel Human Protein Module for NGS | Nanostring Technologies, GMX-PROMOD-NGS-HPDP-12 |
| GeoMx Alzheimer's Pathology Extended Panel Human Protein Module for NGS | Nanostring Technologies, GMX-PROMOD-NGS-HADEP-12 |
| GeoMx Cell Death Panel Human Protein Module for NGS | Nanostring Technologies, GMX-PROMOD-NGS-HCD-12 |
| GeoMx Glial Cell Subtyping Panel Human Protein Module for NGS | Nanostring Technologies, GMX-PROMOD-NGS-HGCS-12 |
| GeoMx Autophagy Panel Human Protein Module for NGS | Nanostring Technologies, GMX-PROMOD-NGS-HA-12 |
| GeoMx Mouse Protein Core for NGS | Nanostring Technologies, GMX-PROCO-NGS-MCORE-12 |
| GeoMx Immune Cell Typing Panel Mouse Protein Module for NGS | Nanostring Technologies, GMX-PROMOD-NGS-MICT-12 |
| GeoMx IO Drug Target Panel Mouse Protein Module for NGS | Nanostring Technologies, GMX-PROMOD-NGS-MIODT-12 |
| GeoMx Immune Activation Status Panel Mouse Protein Module for NGS | Nanostring Technologies, GMX-PROMOD-NGS-MIAS-12 |
| GeoMx Pan-Tumor Panel Mouse Protein Module for NGS | Nanostring Technologies, GMX-PROMOD-NGS-MPT-12 |
| GeoMx Myeloid Panel Mouse Protein Module for NGS | Nanostring Technologies, GMX-PROMOD-NGS-MMY-12 |
| GeoMx MAPK Signaling Panel Mouse Protein Module for NGS | Nanostring Technologies, GMX-PROMOD-NGS-MMAPK-12 |
| GeoMx PI3K/AKT Signaling Panel Mouse Protein Module for NGS | Nanostring Technologies, GMX-PROMOD-NGS-MPI3K-12 |
| GeoMx Neural Cell Typing Panel Mouse Protein Module for NGS | Nanostring Technologies, GMX-PROMOD-NGS-MNCT-12 |
| GeoMx Alzheimer's Pathology Panel Mouse Protein Module for NGS | Nanostring Technologies, GMX-PROMOD-NGS-MADP-12 |
| GeoMx Parkinson's Pathology Panel Mouse Protein Module for NGS | Nanostring Technologies, GMX-PROMOD-NGS-MPDP-12 |
| GeoMx Alzheimer's Pathology Extended Panel Mouse Protein Module for NGS | Nanostring Technologies, GMX-PROMOD-NGS-MADEP-12 |
| GeoMx Cell Death Panel Mouse Protein Module for NGS | Nanostring Technologies, GMX-PROMOD-NGS-MCD-12 |
| GeoMx Glial Cell Subtyping Panel Mouse Protein Module for NGS | Nanostring Technologies, GMX-PROMOD-NGS-MGCS-12 |
| GeoMx Autophagy Panel Mouse Protein Module for NGS | Nanostring Technologies, GMX-PROMOD-NGS-MA-12 |
| GeoMx Protein Slide Prep Kit for FFPE | Nanostring Technologies, GMX -PREP-PRO-FFPE-12 |
| GeoMx Hyb Code Pack Protein | Nanostring Technologies, GMX-PRO-HYB-96 |
| GeoMx DSP Collection Plate | Nanostring Technologies, GMX-DSP-COLL-PLT-4 |
| GeoMx DSP Instrument Buffer Kit | Nanostring Technologies, GMX-DSP-BUFF-KIT |
| GeoMx Seq Code Pack | Nanostring Technologies, GMX-NGS-SEQ-AB |
| GeoMx Human Whole Transcriptome Atlas Human RNA for Illumina Systems | Nanostring Technologies, GMX-RNA-NGS-HuWTA-4 |
| GeoMx RNA Slide Prep Kit for FFPE | Nanostring Technologies, GMX-PREP-RNA-FFPE-12 |
| GeoMx Cancer Transcriptome Atlas Human RNA for Illumina Systems | Nanostring Technologies, GMX-RNA-NGS-CTA-4 |
| GeoMx Mouse Whole Transcriptome Atlas Mouse RNA for Illumina Systems | Nanostring Technologies, GMX-RNA-NGS-MsWTA-4 |

**Supplementary Table S3:** A list of Human protein targets by module.

| NGS Human Panel | Target |
|---|---|
| GeoMx Human Immune Activation Status Panel | CD127 |
| | CD25 |
| | CD27 |
| | CD44 |
| | CD45RO |
| | CD80 |
| | ICOS |
| | PD-1 |
| | PD-L1 |
| | PD-L2 |
| GeoMx Human NGS Cell Death Panel | BAD |
| | BCL6 |
| | BCLXL |
| | BIM |
| | CD95/Fas |
| | Cleaved Caspase 9 |
| | GZMA |
| | NF-1 (Neurofibromin-1) |
| | p53 |
| | PARP |
| GeoMx Human NGS Immune Cell Typing Panel | CD20 |
| | CD3 |
| | CD34 |
| | CD4 |
| | CD56 |
| | CD66b |
| | CD8 |
| | Fibronectin |
| | FOXP3 |
| | GZMB |
| GeoMx Human NGS IO Drug Target Panel | 4-1BB |
| | B7-H3 |
| | CTLA4 |
| | GITR |
| | IDO1 |
| | LAG3 |
| | OX40L |
| | STING |
| | Tim-3 |
| | VISTA |
| GeoMx Human NGS MAPK Signaling Panel | BRAF |
| | EGFR |
| | JNK (phospho T183/Y185) |
| | MEK1 (phospho S217/S221) |
| | P38 (phospho T180/Y182) |
| | p44/42 MAPK ERK1/2 |
| | p44/42 MAPK ERK1/2 (phospho T202/Y204) |
| | P90RSK (phospho T359/S363) |
| | pan-RAS |
| GeoMx Human NGS Myeloid Panel | ARG1 |
| | CD11b |
| | CD11c |
| | CD14 |
| | CD163 |
| | CD39 |
| | CD40 |
| | CD68 |
| | HLA-DR |
| GeoMx Human NGS Pan-Tumor Panel | Bcl-2 |
| | EpCAM |
| | ER-alpha |
| | Her2 |
| | MART1 |
| | NY-ESO-1 |
| | PAN-CK |
| | PR |
| | PTEN |
| | SMA |
| GeoMx Human NGS PI3K/AKT Signaling Panel | AKT (phospho S473) |
| | GSK3 (phospho S9) |
| | GSK3αβ (phospho S21/S9) |
| | INPP4B |
| | MET |
| | Pan-AKT |
| | PLCG1 |
| | P-RAS-40 (phopho T246) |
| | TUBERIN (phospho T1462) |

| NGS Human Panel | Target |
|---|---|
| GeoMx Human NGS Alzheimer's Extended Pathology Panel | ADAM10 |
| | BACE1 |
| | IDE |
| | Neprilysin |
| | Neurogranin |
| | PSEN1 |
| | p-Tau (S199) |
| | p-Tau (S396) |
| | p-Tau (T214) |
| | p-Tau (T231) |
| GeoMx Human NGS Alzheimer's Pathology Panel | Amyloid Precursor Protein |
| | Amyloid-Beta 1-40 |
| | Amyloid-Beta 1-42 |
| | APOE |
| | P2RX7 |
| | Phospho-Tau (S404) |
| | Phospho-Tdp-43 (S409/S410) |
| | Tau |
| | Tdp-43 |
| | Ubiquitin |
| GeoMx Human NGS Autophagy Panel | ATG12 |
| | ATG5 |
| | BAG3 |
| | GBA |
| | HSC70 |
| | LAMP2A |
| | LC3B |
| | P62 |
| | TFEB |
| | VPS35 |
| GeoMx Human NGS Glial Cell Subtyping Panel | C4B |
| | CD9 |
| | Clec7a |
| | CSF1R |
| | Ctsd |
| | Emp1 |
| | GPNMB |
| | Mertk |
| | S100B |
| | VIM |
| GeoMx Human NGS Neural Cell Typing Panel | GFAP |
| | IBA1 |
| | MAP2 |
| | Myelin basic protein |
| | NeuN |
| | Neurofilament light |
| | Olig2 |
| | P2ry12 |
| | Synaptophysin |
| | TMEM119 |
| GeoMx Human NGS Parkinson's Pathology Panel | Alpha-synuclein |
| | ApoA-I |
| | Calbindin |
| | FUS |
| | LRRK2 |
| | Park5 |
| | Park7 |
| | Phospho-Alpha-synuclein (S129) |
| | PINK1 |
| | Tyrosine Hydroxylase |
| GeoMx Human NGS Core | Beta-2-microglobulin |
| | CD31 |
| | CD45 |
| | GAPDH |
| | Histone H3 |
| | Ki-67 |
| | Ms IgG1 |
| | Ms IgG2a |
| | Rb IgG |
| | S6 |

**Supplementary Table S4:** A list of Mouse protein targets by module.

| NGS Mouse Panel | Target |
|---|---|
| GeoMx Mouse Immune Activation Status Panel | CD127 |
| | CD27 |
| | CD40L |
| | CD44 |
| | CD86 |
| | ICOS |
| | PD-1 |
| | PD-L1 |
| GeoMx Mouse NGS Cell Death Panel | BAD |
| | BCLXL |
| | BIM |
| | Cleaved Caspase 3 |
| | gamma-H2AX |
| | NF-1 (Neurofibromin-1) |
| | p21 |
| | p53 |
| | PARP |
| | Perforin |
| GeoMx Mouse NGS Immune Cell Typing Panel | BatF3 |
| | CD19 |
| | CD28 |
| | CD3 |
| | CD34 |
| | CD4 |
| | CD8 |
| | Fibronectin |
| | FOXP3 |
| | GZMB |
| GeoMx Mouse NGS IO Drug Target Panel | B7-H3 |
| | CTLA4 |
| | GITR |
| | LAG3 |
| | OX40L |
| | Tim-3 |
| | VISTA |
| GeoMx Mouse NGS MAPK Signaling Panel | BRAF |
| | EGFR |
| | JNK (phospho T183/Y185) |
| | MEK1 |
| | MEK1 (phospho S217/S221) |
| | P38 |
| | p44/42 MAPK ERK1/2 |
| | p44/42 MAPK ERK1/2 (phospho T202/Y204) |
| | P90RSK (phospho T359/S363) |
| | pan-RAS |
| GeoMx Mouse NGS Myeloid Panel | CD11b |
| | CD11c |
| | CD14 |
| | CD163 |
| | CD39 |
| | CD40 |
| | CD68 |
| | F4/80 |
| | Ly6G/Ly6C |
| | MHC II |
| GeoMx Mouse NGS Pan-Tumor Panel | AhR |
| | AR |
| | EpCAM |
| | ER-alpha |
| | Her2 |
| | IFNGR |
| | PAN-CK |
| | Pmel17 |
| | SMA |
| GeoMx Mouse NGS PI3K/AKT Signaling Panel | AKT (phospho S473) |
| | AMPK-alpha_pThr172 |
| | GSK3αβ (phospho S21/S9) |
| | MET |
| | Pan-AKT |
| | PLCG1 |
| | P-RAS-40 (phopho T246) |
| | S6_pS235/236 |

| NGS Mouse Panel | Target |
|---|---|
| GeoMx Mouse NGS Alzheimer's Extended Pathology Panel | BACE1 |
| | IDE |
| | Neprilysin |
| | Neurogranin |
| | PSEN1 |
| | p-Tau (S199) |
| | p-Tau (S396) |
| | p-Tau (T214) |
| | p-Tau (T231) |
| GeoMx Mouse NGS Alzheimer's Pathology Panel | Amyloid Precursor Protein |
| | Amyloid-Beta 1-42 |
| | APOE |
| | P2RX7 |
| | Phospho-Tau (S404) |
| | Tau |
| | Tdp-43 |
| | Ubiquitin |
| GeoMx Mouse NGS Autophagy Panel | ATG12 |
| | ATG5 |
| | BAG3 |
| | Beclin-1 |
| | LC3B |
| | P62 |
| | PLA2G6 |
| | TFEB |
| | ULK1 |
| | VPS35 |
| GeoMx Mouse NGS Glial Cell Subtyping Panel | Aldh1l1 |
| | CD9 |
| | CSF1R |
| | Ctsd |
| | GPNMB |
| | Mertk |
| | MSR1 |
| | S100B |
| | SPP1 |
| | VIM |
| GeoMx Mouse NGS Neural Cell Typing Panel | GFAP |
| | IBA1 |
| | MAP2 |
| | Myelin basic protein |
| | NeuN |
| | Neurofilament light |
| | Olig2 |
| | Synaptophysin |
| | TMEM119 |
| GeoMx Mouse NGS Parkinson's Pathology Panel | Alpha-synuclein |
| | ApoA-I |
| | Calbindin |
| | LRRK2 |
| | Park5 |
| | Park7 |
| | Phospho-Alpha-synuclein (S129) |
| | PINK1 |
| | Tyrosine Hydroxylase |
| GeoMx Mouse NGS Core | CD31 |
| | CD45 |
| | GAPDH |
| | GFP |
| | Histone H3 |
| | Ki-67 |
| | Rat IgG2a |
| | Rat IgG2b |
| | Rb IgG |
| | S6 |

**Supplementary Table S5:** The 11-cell pellet array (CPA) used in assay development.

| | Cell Line | Tissue | Cell Sex | Source, RRID |
|---|---|---|---|---|
| 1 | COLO201 | Large intestine | Male | (ATCC Cat# CCL-224, RRID:CVCL_F399) |
| 2 | DAUDI | Haematopoietic and lymphoid | Male | (ATCC Cat# CCL-213, RRID:CVCL_0008) |
| 3 | H596 | Lung | Male | (ATCC Cat# HTB178, RRID:CVCL_1571) |
| 4 | HDLM2 | Haematopoietic and lymphoid | Male | (DSMZ Cat# ACC 17, RRID:CVCL_0009) |
| 5 | HEL | Haematopoietic and lymphoid | Male | (ATCC Cat# TIB-180, RRID:CVCL_2481) |
| 6 | HS578T | Breast | Female | (ATCC Cat# HTB-126, RRID:CVCL_0332) |
| 7 | HUT78 | Haematopoietic and lymphoid | Male | (ATCC Cat# TIB161, RRID:CVCL_0337) |
| 8 | MALME3M | Skin | Male | (ATCC Cat# HTB-64, RRID:CVCL_1438) |
| 9 | OPM2 | Haematopoietic and lymphoid | Female | (DSMZ Cat# ACC 50, RRID:CVCL_1625) |
| 10 | THP1 | Haematopoietic and lymphoid | Male | (ATCC Cat# TIB-202, RRID:CVCL_0006) |
| 11 | U118MG | Brain | Male | (ATCC Cat# HTB-15, RRID:CVCL_0633) |

**Supplementary Table S6:** The 45-cell pellet array (CPA) used in assay development.

| CP# | Cell Line | Tissue Origen | GENE, Accession | Treatment | Cell Sex | Source, RRID |
|---|---|---|---|---|---|---|
| 1 | HEK293 Tim3 | Kidney | TIM3, NM_032782.4 | Stable over-expressor in HEK293T (RRID:CVCL_0045) | Female | (Crown Bio Cat# C2021) |
| 2 | HEK293 4-1BB | Kidney | CD137, NM_001561.5 | | Female | (Crown Bio Cat# C2012) |
| 3 | HEK293 PDL1 | Kidney | CD274, NM_014143 | | Female | (Crown Bio Cat# C2011) |
| 4 | HEK293 PDL2 | Kidney | PDL2, NM_025239.3 | | Female | (Crown Bio Cat# C2025) |
| 5 | HEK293 Lag3 | Kidney | LAG3, NM_002286.5 | | Female | (Crown Bio Cat# C2020) |
| 6 | HEK293 GITR | Kidney | GITR, NM_009400.2 | | Female | (Crown Bio Cat# C2011) |
| 7 | HEK293 CTLA4 | Kidney | CTLA4, NM_005214.4 | | Female | (Crown Bio Cat# C2015) |
| 8 | HEK293 PD1 | Kidney | PDCD1, NM_005018 | | Female | (Crown Bio Cat# C2004) |
| 9 | HEK293 ICOS | Kidney | ICOS, NM_012092.3 | | Female | (Crown Bio Cat# C2016) |
| 10 | A431 | Skin | | | Female | (ATCC Cat# CRL1555, RRID:CVCL_0037) |
| 11 | CCRF-CEM | Peripheral blood | | | Female | (ATCC Cat# CCL-119, RRID:CVCL_0207) |
| 12 | 22rv1 | Prostate | | | Male | (ATCC Cat# CRL2505, RRID:CVCL_1045) |
| 13 | H596 | Lung | | | Male | (ATCC Cat# HTB178, RRID:CVCL_1571) |
| 14 | HCC78 | Lung | | | Male | (DSMZ Cat# ACC 563, RRID:CVCL_2061) |
| 15 | HCT 116 | Large intestine | | | Male | (ATCC Cat# CCL-247, RRID:CVCL_0291) |
| 16 | HL-60 | Peripheral blood | | | Female | (ATCC Cat# CCL-240, RRID:CVCL_0002) |
| 17 | HUH7 | Haematopoietic and Lymphoid | | | Male | (ECACC Sigma Cat# 01042712, RRID:CVCL_0336) |
| 18 | Hut78 | Haematopoietic and Lymphoid | | | Male | (ATCC Cat# TIB161, RRID:CVCL_0337) |
| 19 | MDA-MB-468 | Breast | | | Female | (ATCC Cat# HTB-132, RRID:CVCL_0419) |
| 20 | K-562 | Haematopoietic and Lymphoid | | | Female | (ATCC Cat# CCL-243, RRID:CVCL_0004) |
| 21 | OVCAR8 | Ovary | | | Female | (ATCC Cat# HTB-161, RRID:CVCL_1629) |
| 22 | Raji | Haematopoietic and Lymphoid | | | Male | (ATCC Cat# CCL-86, RRID:CVCL_0511) |
| 23 | Ri-1 | Haematopoietic and Lymphoid | | | Female | (DSMZ Cat# ACC 585, RRID:CVCL_1885) |
| 24 | SU-DHL-6 | B-cell Lymphoma | | | Male | (ATCC Cat# CRL-2959, RRID:CVCL_2206) |
| 25 | RPMI-8226 | Haematopoietic and Lymphoid | | | Male | (ATCC Cat# CCL-155, RRID:CVCL_0014) |
| 26 | RAMOS | Haematopoietic and Lymphoid | | | Male | (ATCC Cat# CRL-1923, RRID:CVCL_0597) |
| 27 | SK-BR-3 | Breast | | | Female | (ATCC Cat# HTB-30, RRID:CVCL_0033) |
| 28 | SK-MEL-5 | Skin | | | Female | (ATCC Cat# HTB-70, RRID:CVCL_0527) |
| 29 | SU-DHL-1 | Haematopoietic and Lymphoid | | | Male | (ATCC Cat# CRl-2955, RRID:CVCL_0538) |
| 30 | SU-DHL-4 | B-cell Lymphoma | | | Male | (ATCC Cat# CRl-2957, RRID:CVCL_0539) |
| 31 | SK-MEL-2 | Skin | | | Male | (ATCC Cat# HTB-68, RRID:CVCL_0069) |
| 32 | WSU-NHL | Haematopoietic and Lymphoid | | | Female | (DSMZ Cat# ACC 58, RRID:CVCL_1793) |
| 33 | THP-1 | Peripheral blood | | | Male | (ATCC Cat# TIB-202, RRID:CVCL_0006) |
| 34 | SUP-B15 | Haematopoietic and Lymphoid | | | Male | (ATCC Cat# CRL-1929, RRID:CVCL_0103) |
| 35 | U87-MG | Brain | | | Male | (ATCC Cat# HTB-14, RRID:CVCL_0022) |
| 36 | U251-MG | Brain | | | Male | (ECACC Sigma Cat# 09063001, RRID:CVCL_0021) |
| 37 | NB4 | Haematopoietic and Lymphoid | | | Female | (DSMZ Cat# ACC 207,RRID:CVCL_0005) |
| 38 | BT-474 | Breast | | | Female | (ATCC Cat# HTB-20, RRID:CVCL_0179) |
| 39 | NK-92 | Peripheral blood | | | Male | (DSMZ Cat# ACC-488, RRID:CVCL_2142) |
| 40 | SW48 | Large intestine | | | Female | (ATCC Cat# CCL-231, RRID:CVCL_1724) |
| 41 | DBTRG-05MG | Brain | | | Female | (ATCC Cat# CRL-2020, RRID:CVCL_1169) |
| 42 | NCI-H2228 | Lung | | | Female | (ATCC Cat# CRl-5935, RRID:CVCL_1543) |
| 43 | A431 CA | Skin | | Calyculin A (TOCRIS Cat #. 1336, 100 nM, 30 min) | Female | (ATCC Cat# CRL1555, RRID:CVCL_0037) |
| 44 | SH-SY5Y CA | Brain | | | Female | (DSMZ Cat# ACC 209, RRID:CVCL_0019) |
| 45 | SKBR3 PI | Breast | | | Female | (ATCC Cat# HTB-30, RRID:CVCL_0033) |
