# Supplementary material for: Ultra High-plex Spatial Proteogenomic Investigation of Giant Cell Glioblastoma Multiforme Immune Infiltrates Reveals Distinct Protein and RNA Expression Profiles
Source: Cancer Res Commun. 2023 May 3;3(5):763–79. doi: 10.1158/2767-9764.CRC-22-0396 (PMC10155752; doi:10.1158/2767-9764.CRC-22-0396)
Supplement: Supplementary Figures S1-S9 — Supplementary Figure S1: Impact of staining order on protein detection. FFPE cell line, A431CA, was stained with GeoMx Protein assays for nCounter readout and mock RNA probe (Buffer R only). (A) Swarm plots showing the distribution of target in relation to SNR. (B) Pearson’s R correlation to the control was calculated for each test condition. (C) The number of targets above the detection threshold, SNR ≥ 3. (D) Summary of the Pearson’s R correlation and percent decrease in detectable targets for each condition compared to the control. Supplementary Figure S2: Assessment of epitope retrieval conditions on the performance of spatial proteogenomic assay in relation to CTA control. A 11-core FFPE cell pellet array (CPA) sections, pretreated under basic or slightly acidic conditions and 1 µg/mL proteinase K, were stained with the GeoMx Human CTA and 59-plex GeoMx Human NGS Protein panels. CPAs stained with single analyte GeoMx Human CTA under standard assay conditions was used as the RNA control. Circular ROIs of 200 µm diameter were selected for detailed molecular profiling with the GeoMx DSP. The signal was averaged across replicate ROIs and the SNR was calculated. Plots represent the correlation of log2-transformed SNR for CTA under (A) slightly acidic HIER and (B) basic HIER. (C) Correlation of the RNA control and proteogenomic assay to the CCLE RNAseq database. (D) Calculated true positive rate (TPR) and false positive rate (FPR) for CTA. Supplementary Figure S3: Assessment of epitope retrieval conditions on the performance of spatial proteogenomic assay in relation to protein control. A 11-core FFPE cell pellet array (CPA) sections, pretreated under basic or slightly acidic conditions and 1 µg/mL proteinase K, were stained with the GeoMx Human CTA and 59-plex GeoMx Human NGS Protein panels. CPAs stained with single-analyte 59-plex GeoMx Human NGS Protein panels under standard assay conditions was used as the protein control. Circular ROIs of 200 µm diameter were se [file crc-22-0396-s02.pdf]

Supplementary Figures

Supplementary Figure S1

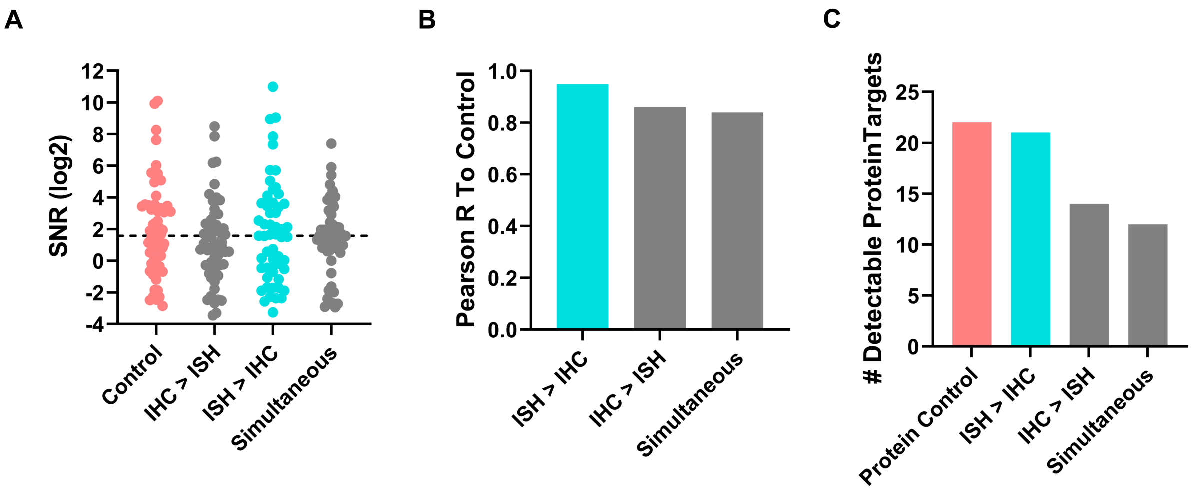

D

Staining Order	Pearson's Correlation to Protein Control	Decrease in Detectable Targets (%)*
Mock RNA > Protein (ISH > IHC)	0.9524	5
Protein > Mock RNA (IHC > ISH)	0.8629	36
Mock RNA:Protein (Simultaneous)	0.8359	45

Supplementary Figure S1: Impact of staining order on protein detection. FFPE cell line, A431CA, was stained with GeoMx Protein assays for nCounter readout and mock RNA probe (Buffer R only). (**A**) Swarm plots showing the distribution of target in relation to SNR. (**B**) Pearson's *R* correlation to the control was calculated for each test condition. (**C**) The number of targets above the detection threshold, SNR $\geq$ 3. (**D**) Summary of the Pearson's *R* correlation and percent decrease in detectable targets for each condition compared to the control.

Supplementary Figure S2

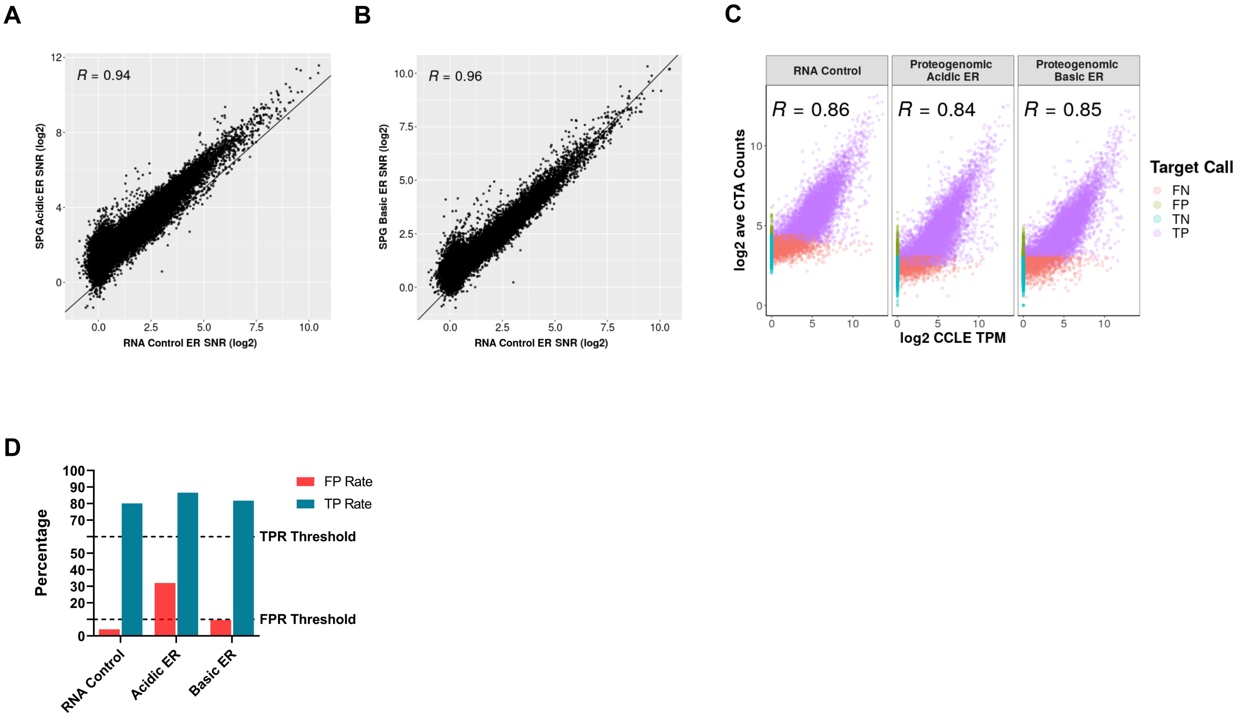

Supplementary Figure S2: Assessment of epitope retrieval conditions on the performance of spatial proteogenomic assay in relation to CTA control. A 11-core FFPE cell pellet array (CPA) sections, pretreated under basic or slightly acidic conditions and 1 µg/mL proteinase K, were stained with the GeoMx Human CTA and 59-plex GeoMx Human NGS Protein panels. CPAs stained with single analyte GeoMx Human CTA under standard assay conditions was used as the RNA control. Circular ROIs of 200 µm diameter were selected for detailed molecular profiling with the GeoMx DSP. The signal was averaged across replicate ROIs and the SNR was calculated. Plots represent the correlation of log2-transformed SNR for CTA under (**A**) slightly acidic HIER and (**B**) basic HIER. (**C**) Correlation of the RNA control and proteogenomic assay to the CCLE RNAseq database. (**D**) Calculated true positive rate (TPR) and false positive rate (FPR) for CTA.

Supplementary Figure S3

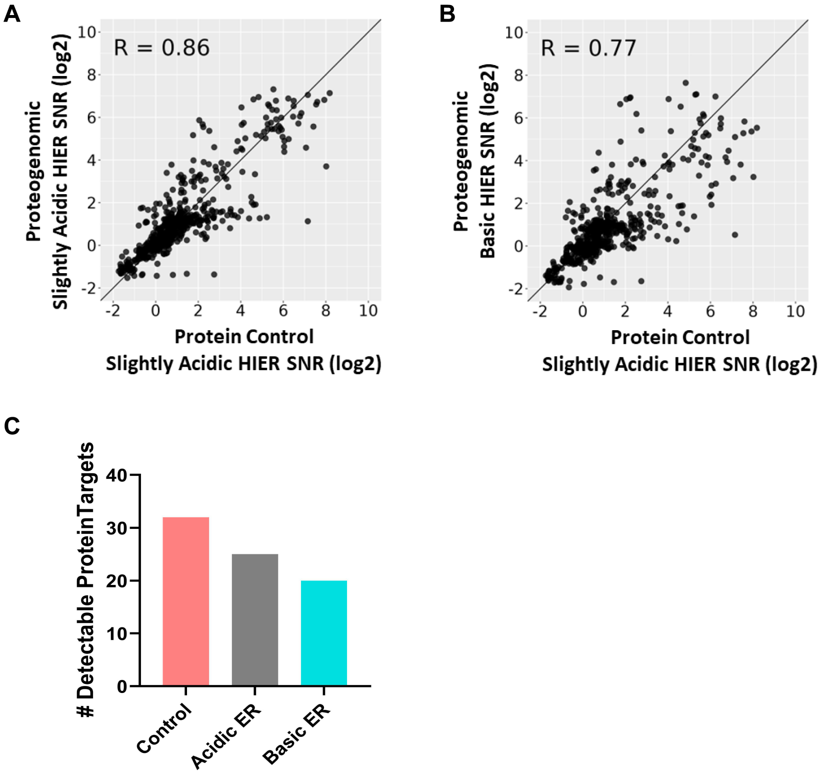

Supplementary Figure S3: Assessment of epitope retrieval conditions on the performance of spatial proteogenomic assay in relation to protein control. A 11-core FFPE cell pellet array (CPA) sections, pretreated under basic or slightly acidic conditions and 1 µg/mL proteinase K, were stained with the GeoMx Human CTA and 59-plex GeoMx Human NGS Protein panels. CPAs stained with single-analyte 59-plex GeoMx Human NGS Protein panels under standard assay conditions was used as the protein control. Circular ROIs of 200 µm diameter were selected for detailed molecular profiling with the GeoMx DSP. The signal was averaged across replicate ROIs and the SNR was calculated. Plots represent the correlation of log2-transformed SNR for protein analyte under (**A**) slightly acidic HIER and (**B**) basic HIER. (**C**) The number of protein targets above detection threshold, SNR $\geq$ 3.

Supplementary Figure S4

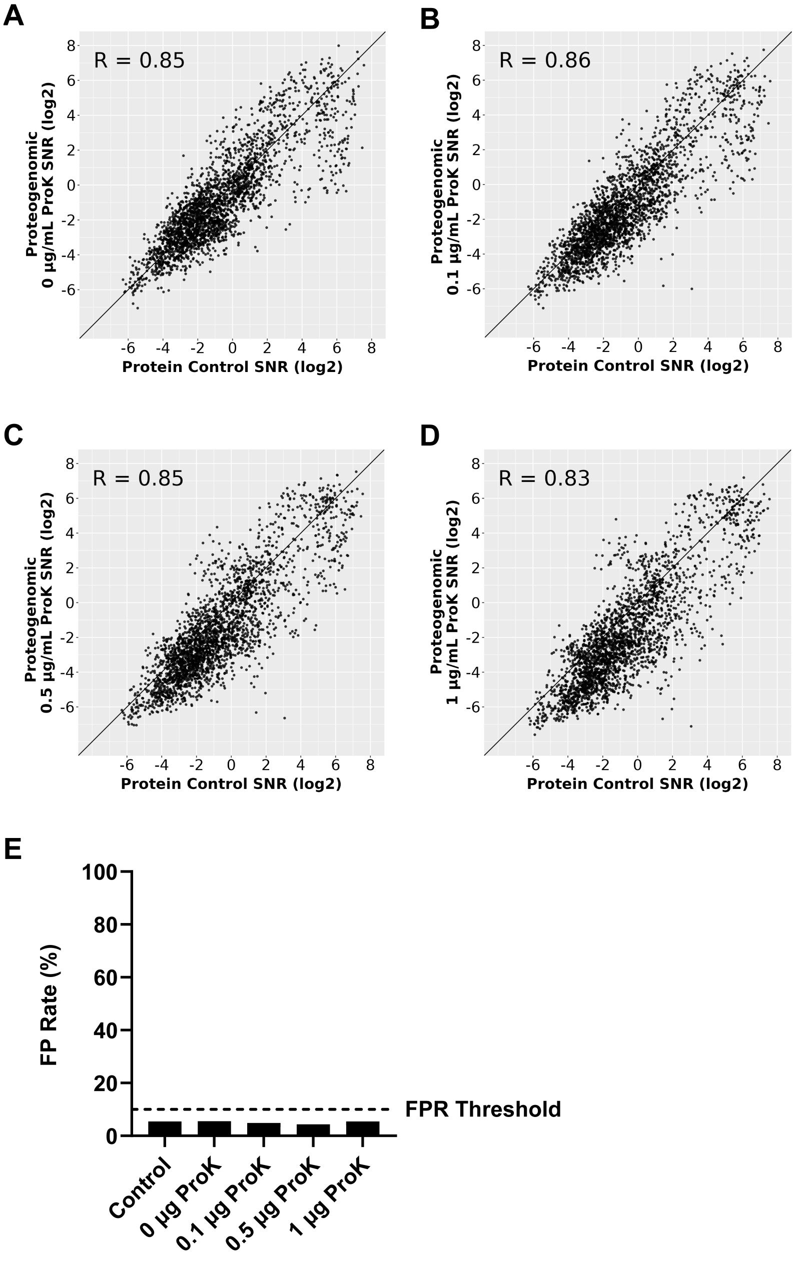

Supplementary Figure S4: Assessment of varying proteinase K (ProK) on assay performance with respect to protein analyte. FFPE sections of a 45-cell pellet array was treated under basic HIER followed by varying concentration of ProK. Pretreated slides were stained with GeoMx Human WTA and 59-plex GeoMx Human NGS Protein panels. CPAs stained with single-analyte 59-plex GeoMx Human NGS Protein panels under standard assay conditions was used as the protein control. Circular ROIs of 200 µm diameter were selected for detailed molecular profiling with the GeoMx DSP. The signal was averaged across replicate

ROIs and the SNR was calculated. Plots represent the correlation of log2-transformed SNR between the protein control and proteogenomic assay (**A**) 0 µg/mL, (**B**) 0.1 µg/mL, (**C**) 0.5 µg/mL, and (**D**) 1µg/mL ProK. (**E**) Calculated false positive rate (FPR) and of protein targets for each test condition. Dotted line specifies an SNR of 3.

Supplementary Figure S5

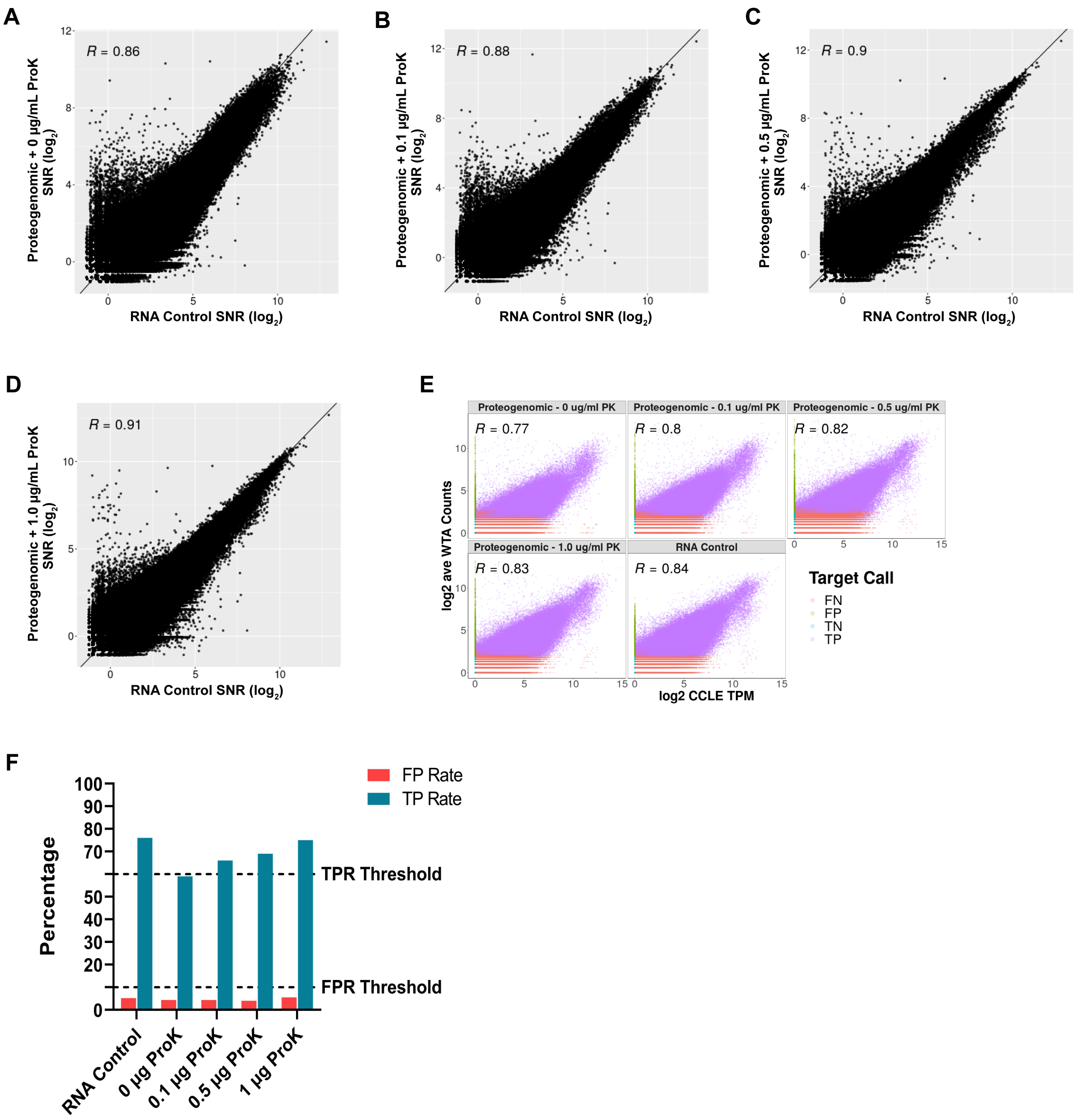

Supplementary Figure S5: Assessment of varying proteinase K (ProK) on assay performance with respect to RNA analyte. FFPE sections of a 45-cell pellet array was treated under basic HIER followed by varying concentration of ProK. Pretreated slides were stained with the GeoMx Human WTA and the 59-plex GeoMx Human NGS Protein panels. CPAs stained with the single-analyte GeoMx Human WTA under standard assay conditions was used as the RNA control. Circular ROIs of 200 µm diameter were selected for detailed molecular profiling with the GeoMx DSP. The signal was averaged across replicate ROIs and the SNR was calculated. Plots represent the correlation of log2-transformed SNR between the RNA control and proteogenomic assay (**A**) 0 µg/mL, (**B**) 0.1 µg/mL, (**C**) 0.5 µg/mL, and (**D**) 1 µg/mL ProK. (**E**) Whole slide correlation between the CCLE database and the proteogenomic assay pretreated under varying concentration of ProK and RNA control. (**F**) Calculated false positive rate (FPR) and true positive rate (TPR) for each of the test conditions.

Supplementary Figure S6

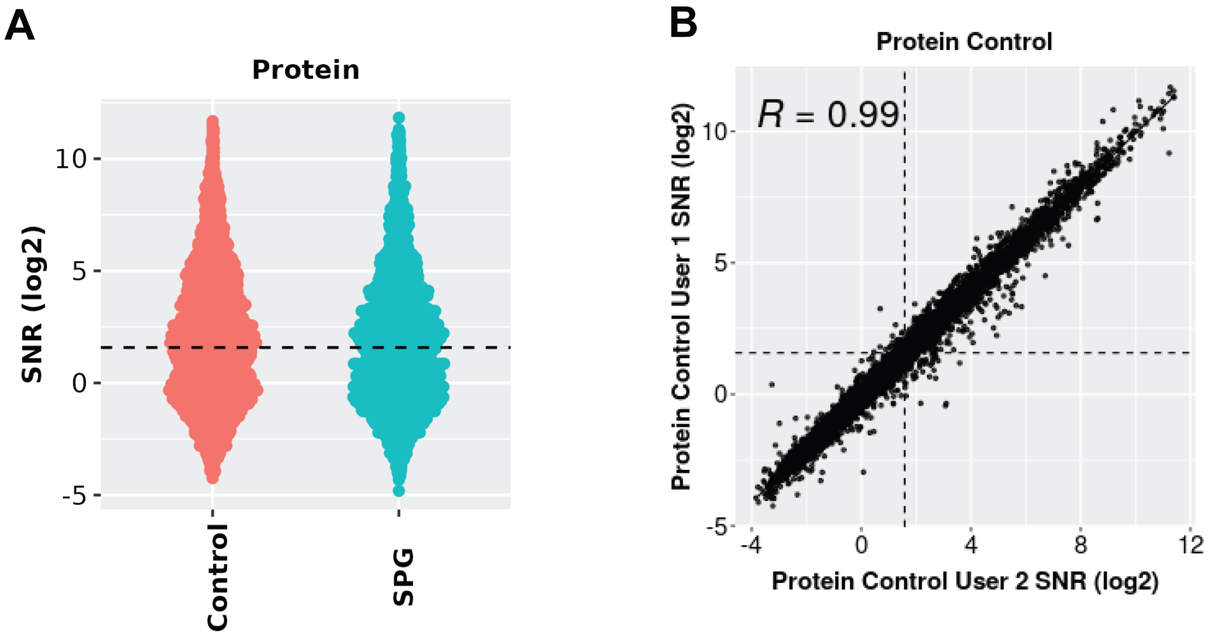

Supplementary Figure S6: Assessment of assay performance for protein analyte. (**A**) Violin plots of the distribution of SNR counts for the proteogenomic assay in comparison to the protein control. (**B**) User-to-user and instrument-to-instrument reproducibility for the single analyte protein control.

Supplementary Figure S7

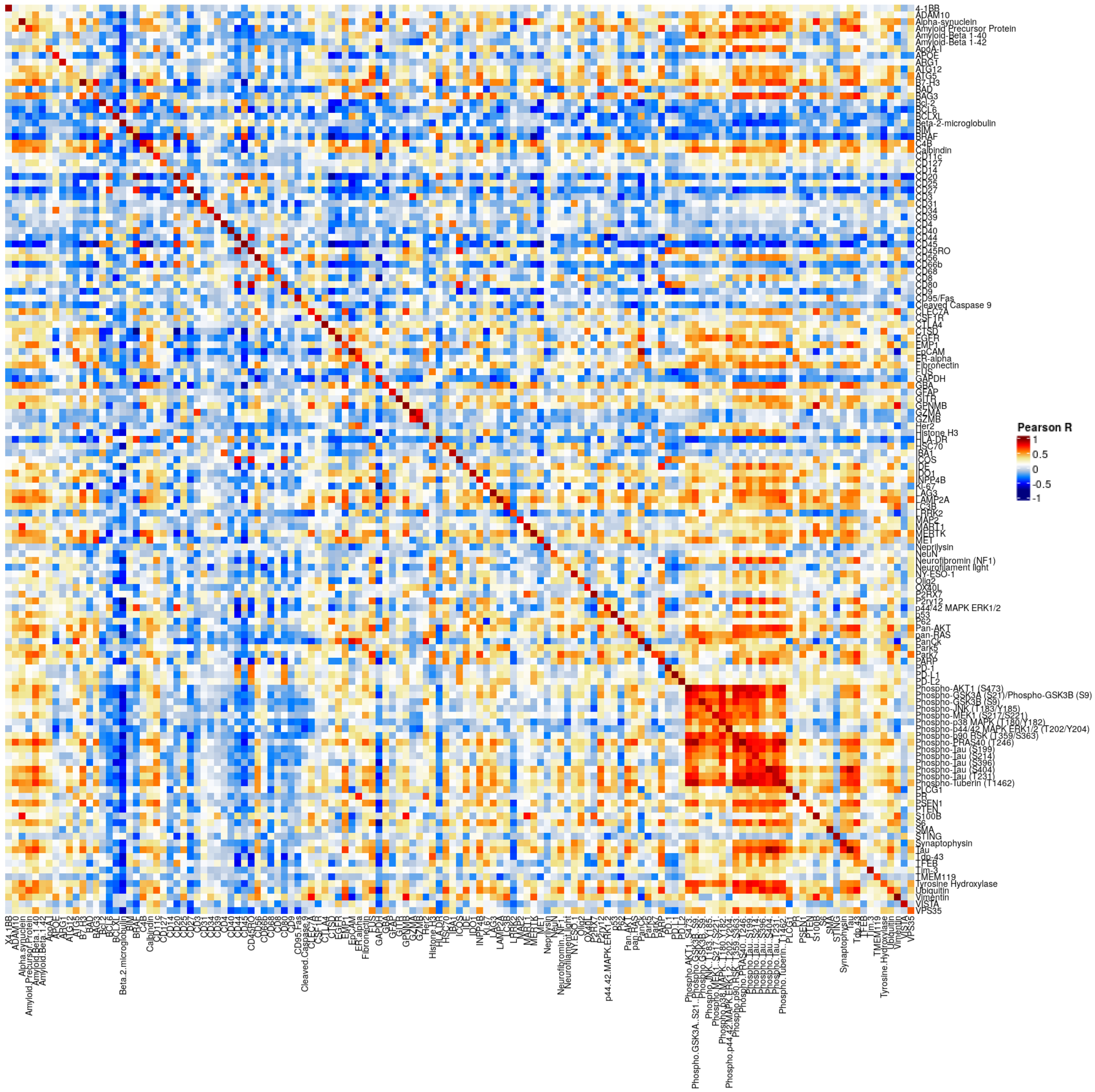

Supplementary Figure S7: Target to target comparison of Protein Control to proteogenomic protein data. For protein targets with SNR ≥ 3, the Pearson's *R* between each protein target from the Protein Control slide were calculated against all targets in the spatial proteogenomic slide. Heatmap of *R* values are displayed.

Supplementary Figure S8

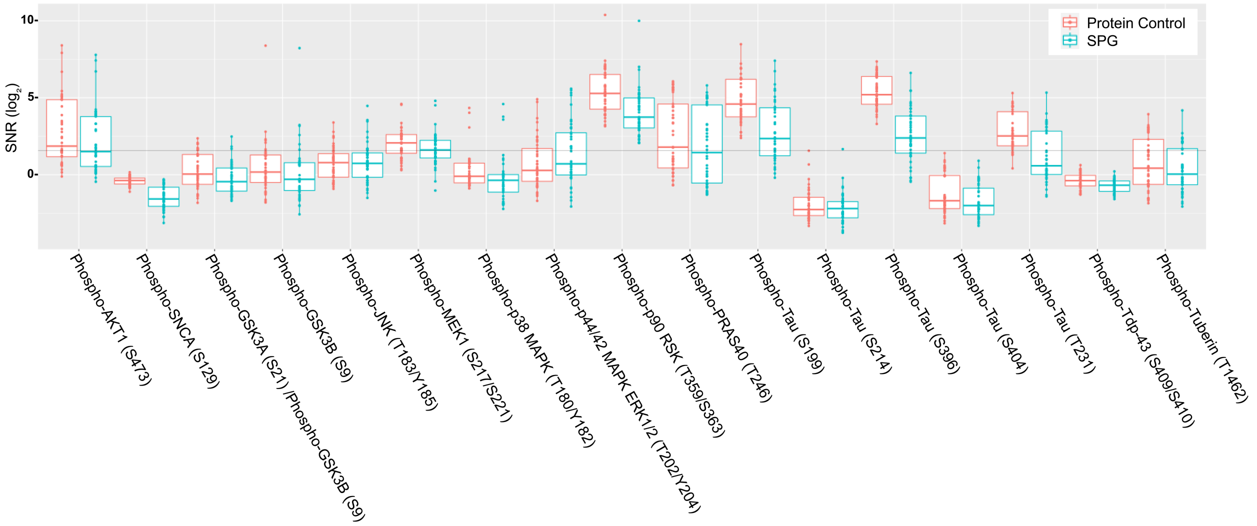

Supplementary Figure S8: Box plot of the signal-to-noise (SNR) of 17 phospho-specific antibodies under standard and proteogenomic conditions for two separate users. Antibody performance was carried out on a 45 CPA which contained cell lines (SKBR3, A431 and SH-SY5Y) that had been treated with a phosphatase inhibitor. For each matching cell line, the Pearson's R was calculated between the two workflows.

Supplementary Figure S9

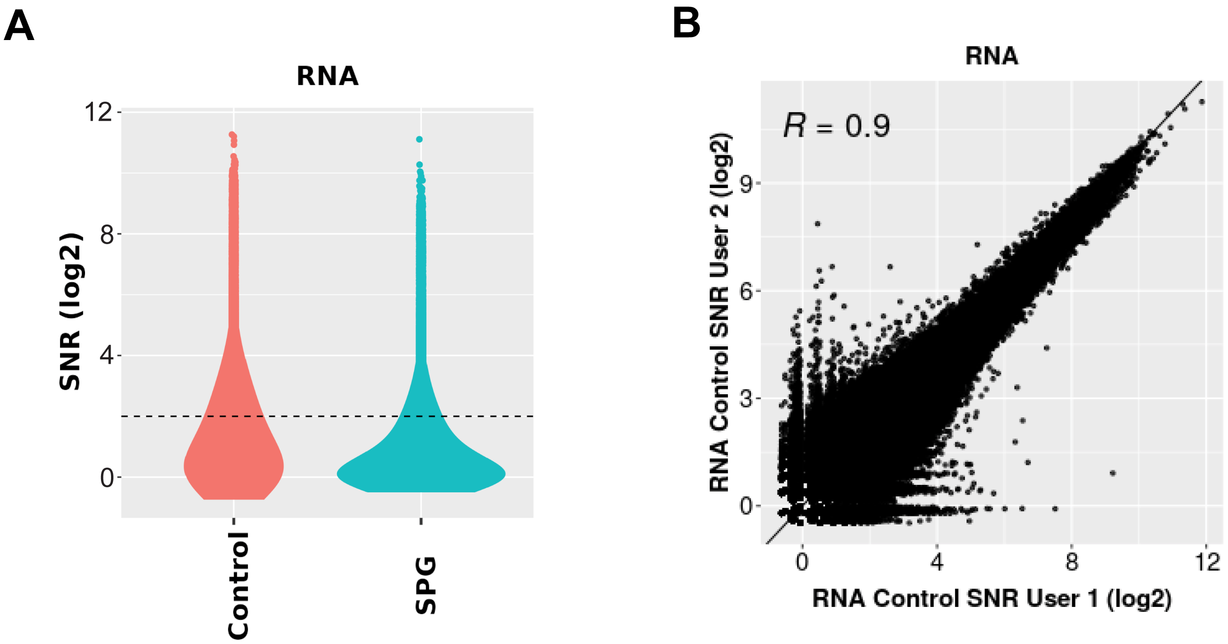

Supplementary Figure S9: Assessment of assay performance for RNA analyte. (**A**) Violin plots of the distribution of SNR counts for the proteogenomic assay in comparison to the RNA control. (**B**) User-to-user and instrument-to-instrument reproducibility for the single analyte RNA control.
