# Supplementary material for: Ultra High-plex Spatial Proteogenomic Investigation of Giant Cell Glioblastoma Multiforme Immune Infiltrates Reveals Distinct Protein and RNA Expression Profiles
Source: Cancer Res Commun. 2023 May 3;3(5):763–79. doi: 10.1158/2767-9764.CRC-22-0396 (PMC10155752; doi:10.1158/2767-9764.CRC-22-0396)
Supplement: Supplementary Figures S10-S15 — Supplementary Figure S10: Representative images of CRC sample used in the assessment of spatial proteogenomic data quality. FFPE colorectal cancer (CRC) sections (A) (zoomed (B)) were stained with the GeoMx NGS Human Protein modules (147-plex), WTA, and antibodies against CD45 (Immune; left panel) and PanCK (Tumor; right panel). Supplementary Figure S11: ROI-to-ROI comparison of the proteogenomic data to the single analyte controls. CRC FFPE sections were stained with 147-plex GeoMx NGS human Protein modules, WTA, and antibodies against PanCK (Tumor) and CD45 (Immune). The Pearson’s R was calculated between each ROI from the proteogenomic assay against all ROIs in the single analyte (A) protein and (B) RNA controls. ROIs are colored according to region (immune or tumor). Protein targets with SNR ≥ 3 and top 400 expressing RNA targets with SNR ≥ 4 were used in the analysis. Supplementary Figure S12: Concordance between matching protein and RNA targets. For protein targets with SNR ≥ 3 and the respective RNA target with SNR ≥ 4, a pairwise scatterplot was generated to visualize the concordance between respective analytes. Pearson’s R calculations are shown in each plot. Supplementary Figure S13: Concordance between Proteogenomic RNA and Protein targets above background in immune segments of colorectal cancer (CRC). FFPE section of CRC stained with 147-plex GeoMx NGS Protein modules, WTA, and antibodies against PanCK (Tumor) and CD45 (Immune) using the proteogenomic workflow. Tumor and immune segments were selected based on PanCK and CD45 immunofluorescence, respectively. Pearson’s R was calculated between each detected protein target (SNR ≥ 3) and RNA targets (SNR ≥ 4) within the immune segment. Supplementary Figure S14: Concordance between Proteogenomic RNA and Protein targets above background in tumor segments of colorectal cancer (CRC). FFPE section of CRC stained with 147-plex GeoMx NGS Protein modules, WTA, and antibodies against PanCK (Tumor) and CD45 (Immune) u [file crc-22-0396-s03.pdf]

Supplementary Figure S10

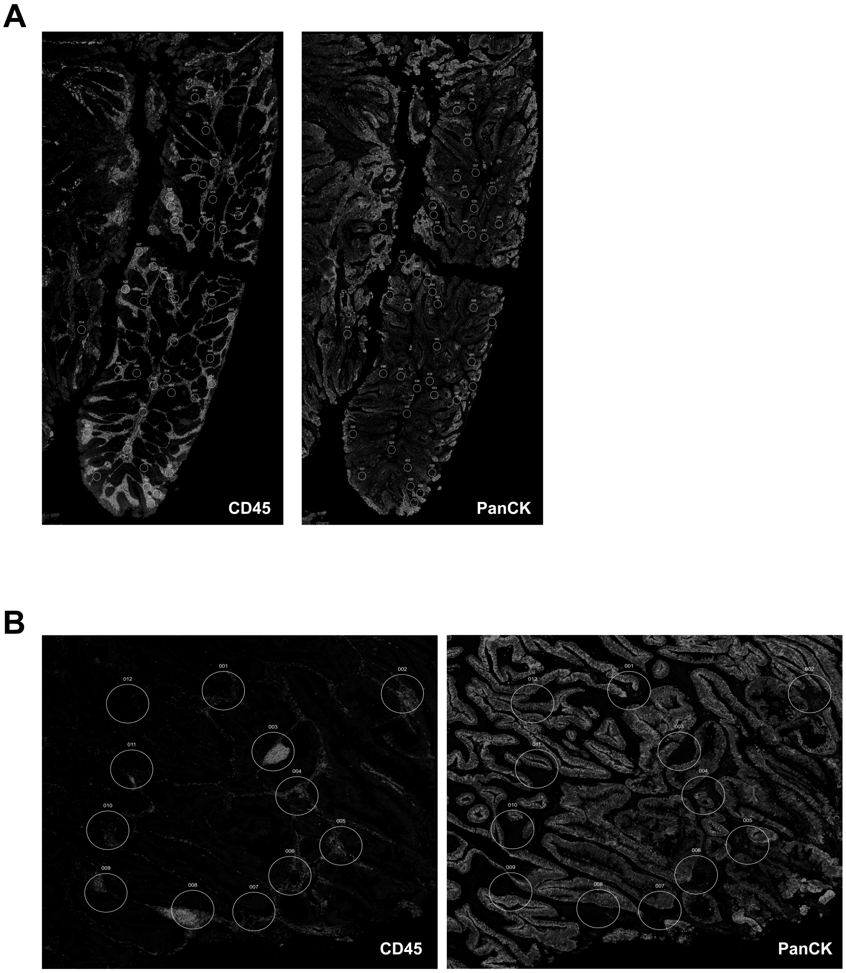

Supplementary Figure S10: Representative images of CRC sample used in the assessment of spatial proteogenomic data quality. FFPE colorectal cancer (CRC) sections (**A**) (zoomed (**B**)) were stained with the GeoMx NGS Human Protein modules (147-plex), WTA, and antibodies against CD45 (Immune; left panel) and PanCK (Tumor; right panel).

Supplementary Figure S11

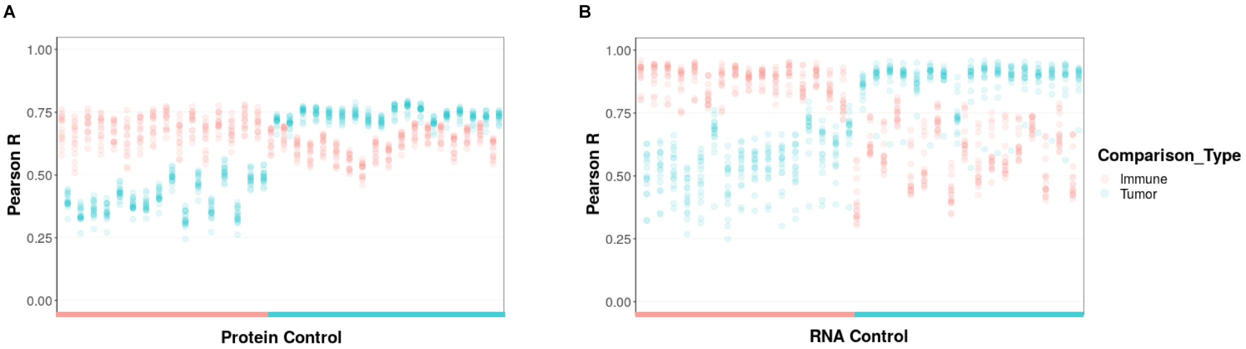

Supplementary Figure S11: ROI-to-ROI comparison of the proteogenomic data to the single analyte controls. CRC FFPE sections were stained with 147-plex GeoMx NGS human Protein modules, WTA, and antibodies against PanCK (Tumor) and CD45 (Immune). The Pearson's *R* was calculated between each ROI from the proteogenomic assay against all ROIs in the single analyte (**A**) protein and (**B**) RNA controls. ROIs are colored according to region (immune or tumor). Protein targets with SNR $\geq$ 3 and top 400 expressing RNA targets with SNR $\geq$ 4 were used in the analysis.

Supplementary Figure S12

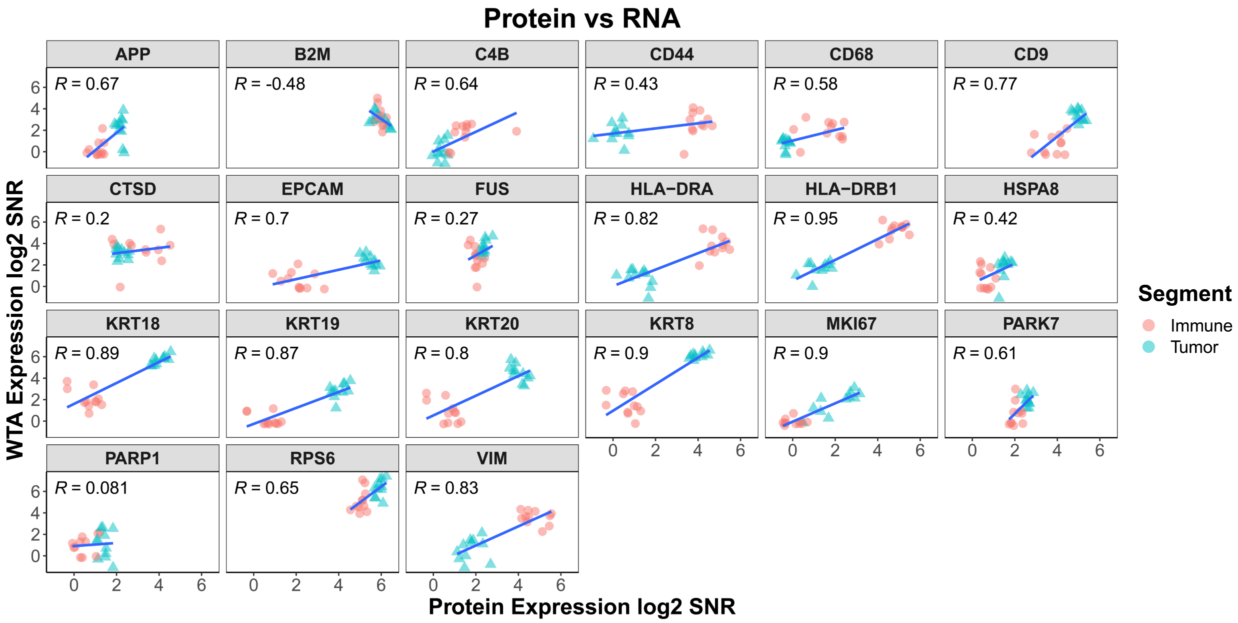

Supplementary Figure S12: Concordance between matching protein and RNA targets. For protein targets with SNR ≥ 3 and the respective RNA target with SNR ≥ 4, a pairwise scatterplot was generated to visualize the concordance between respective analytes. Pearson's *R* calculations are shown in each plot.

Supplementary Figure S13

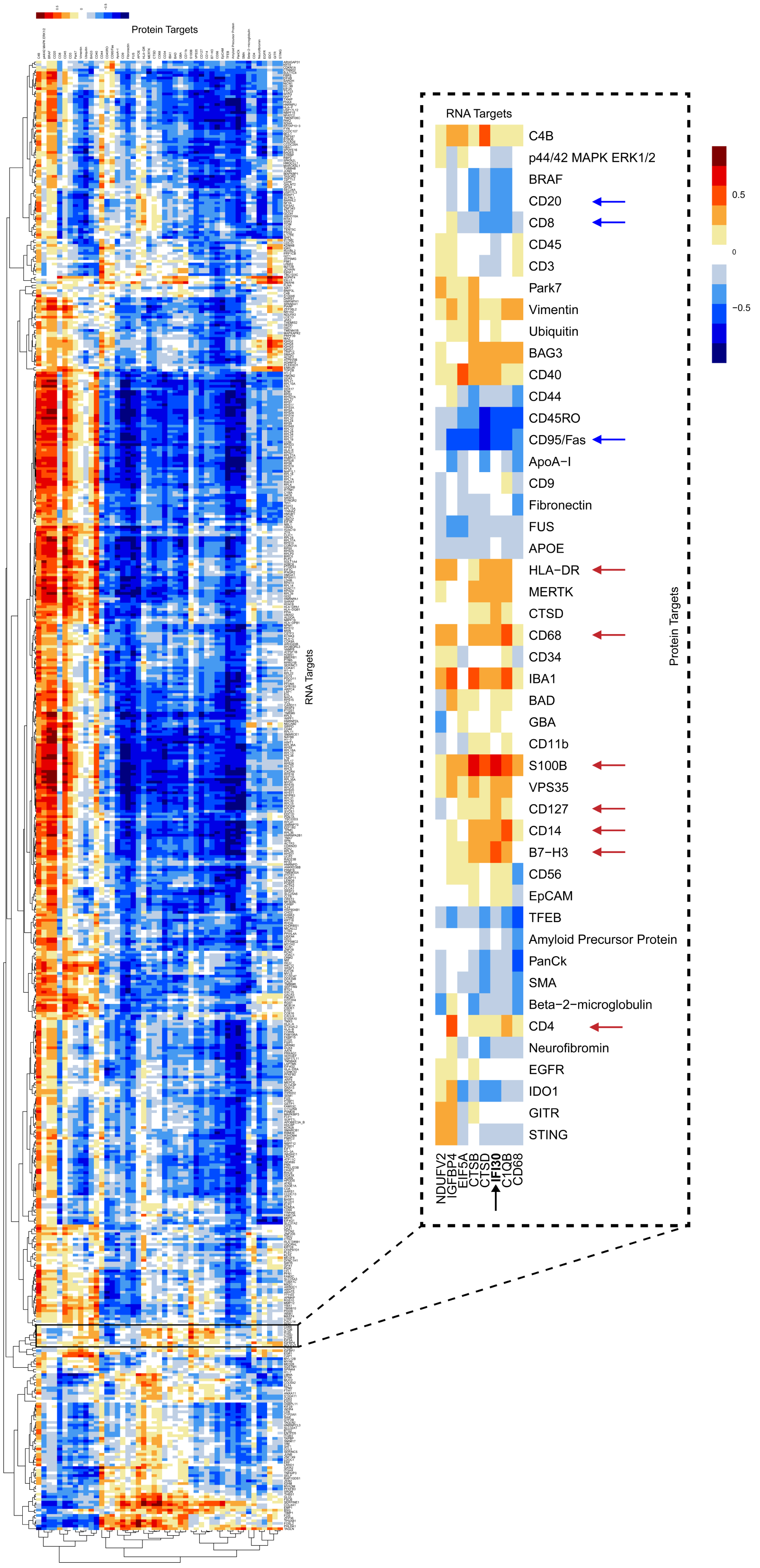

Supplementary Figure S13: Concordance between Proteogenomic RNA and Protein targets above background in immune segments of colorectal cancer (CRC). FFPE section of CRC stained with 147-plex GeoMx NGS Protein modules, WTA, and antibodies against PanCK (Tumor) and CD45 (Immune) using the proteogenomic workflow. Tumor and immune segments were selected based on PanCK and CD45 immunofluorescence, respectively. Pearson's *R* was calculated between each detected protein target (SNR $\geq$ 3) and RNA targets (SNR $\geq$ 4) within the immune segment.

Supplementary Figure S14

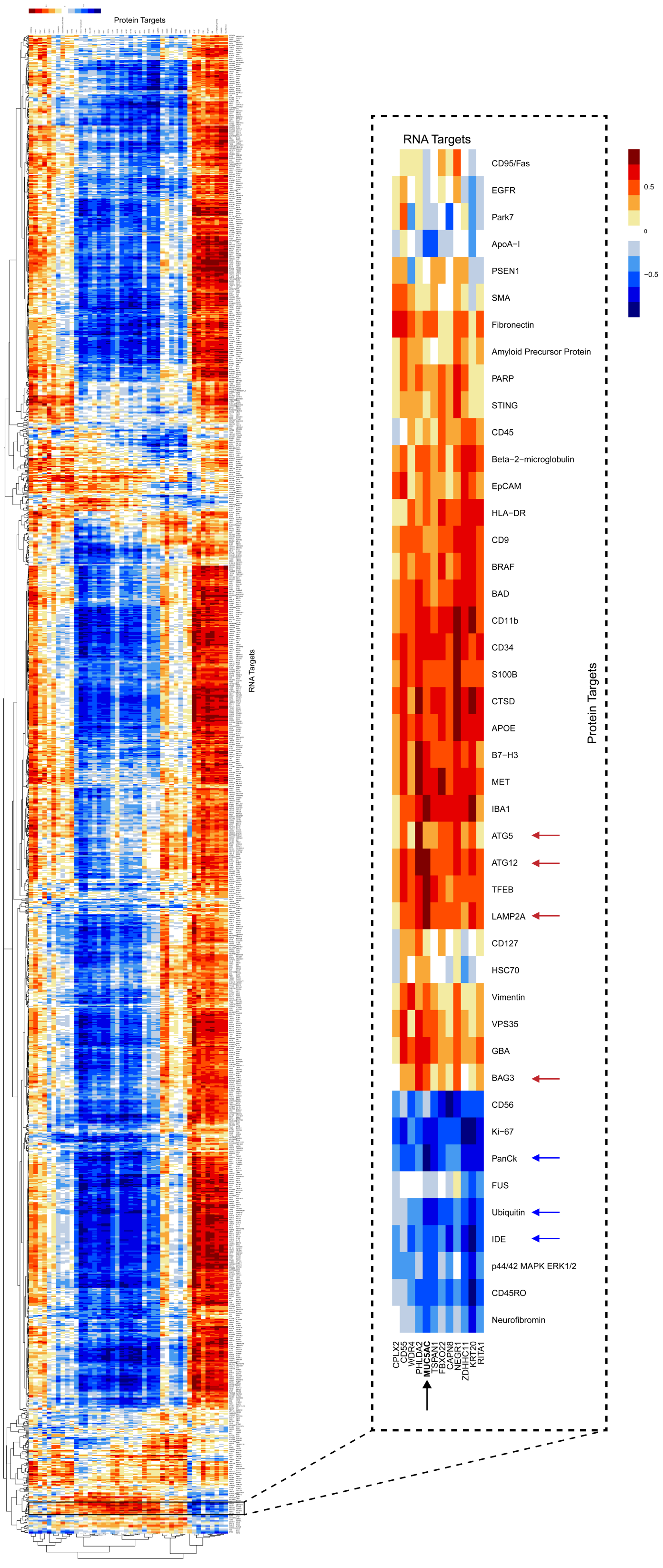

Supplementary Figure S14: Concordance between Proteogenomic RNA and Protein targets above background in tumor segments of colorectal cancer (CRC). FFPE section of CRC stained with 147-plex GeoMx NGS Protein modules, WTA, and antibodies against PanCK (Tumor) and CD45 (Immune) using the proteogenomic workflow. Tumor and immune segments were selected based on PanCK and CD45 immunofluorescence, respectively. Pearson's *R* was calculated between each detected protein target (SNR $\geq$ 3) and RNA targets (SNR $\geq$ 4) within the tumor segment.

Supplementary Figure S15

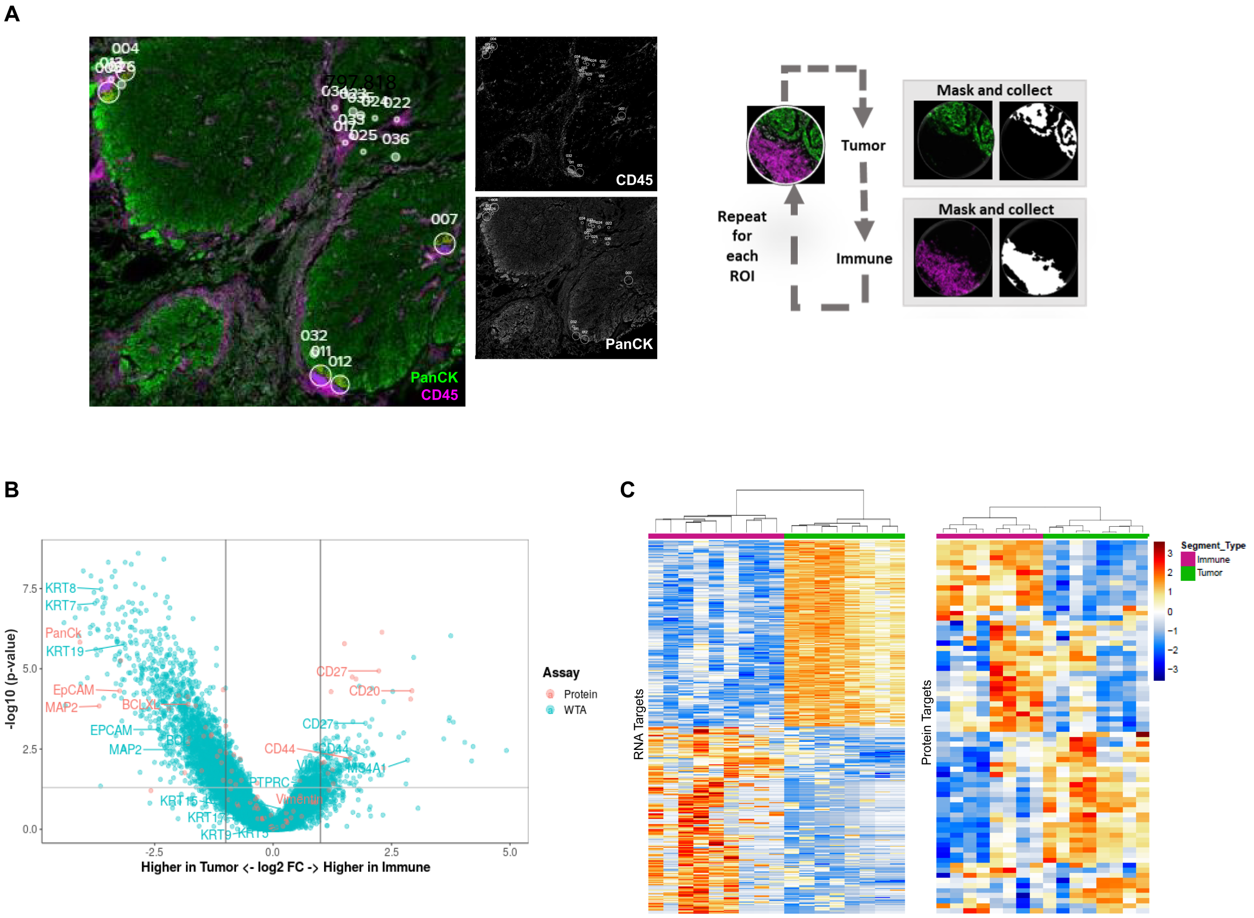

Supplementary Figure S15: Assessment of spatial proteogenomic performance on human NSCLC. NSCLC FFPE sections were stained with 147-plex GeoMx NGS human Protein modules, WTA, and antibodies against PanCK (Tumor) and CD45 (Immune). (**A**) Multiplexed protein and RNA characterization of NSCLC sample with representative colored and gray scaled images highlighting the segmentation of 300 µm circular ROIs into tumor (PanCK$^+$) and immune (CD45$^+$) enriched regions. Segments illuminated in white were collected, black regions were not. Protein and RNA counts were SNR transformed and protein targets with SNR $\geq$ 3 and RNA targets with SNR $\geq$ 4 were used in the analysis. (**B**) Combined volcano plot of protein and RNA expression in NSCLC. All immune segments were compared to all tumor segments for protein and RNA targets above background. A subset of differentially expressed genes are labeled with colors matching their analyte. (**C**) Unsupervised hierarchical clustering of detected RNA (left) and protein (right) targets for NSCLC.
