# Supplementary material for: Ultra High-plex Spatial Proteogenomic Investigation of Giant Cell Glioblastoma Multiforme Immune Infiltrates Reveals Distinct Protein and RNA Expression Profiles
Source: Cancer Res Commun. 2023 May 3;3(5):763–79. doi: 10.1158/2767-9764.CRC-22-0396 (PMC10155752; doi:10.1158/2767-9764.CRC-22-0396)
Supplement: Supplementary Figures S16-S18 — Supplementary Figure S16: Representative grey scale images of used in the assessment of spatial proteogenomic data quality across mouse tissue types. FFPE sections were stained with the GeoMx NGS Mouse Protein modules (137-plex), GeoMx Mm WTA, and antibodies against CD45 (Immune), PanCK (Tumor) and Syto13 (nuclear, DNA). Supplementary Figure S17: ROI-to-ROI comparison of the proteogenomic data to the single analyte controls. Mouse FFPE sections were stained with 137-plex GeoMx NGS Mouse Protein modules and GeoMx Mm WTA. The Pearson’s R was calculated between each ROI from the proteogenomic assay against all ROIs in the single analyte (A) RNA and (B) protein controls. Protein targets with SNR ≥ 3 and RNA targets with SNR ≥ 4 were used in the analysis. Supplementary Figure S18: Expression levels of select matching RNA and protein targets in several mouse tissue types. For protein targets with SNR ≥ 3 and the respective RNA target with SNR ≥ 4, a pairwise scatter plot was generated to visualize the concordance between respective analytes. [file crc-22-0396-s04.pdf]

Supplementary Figure S16

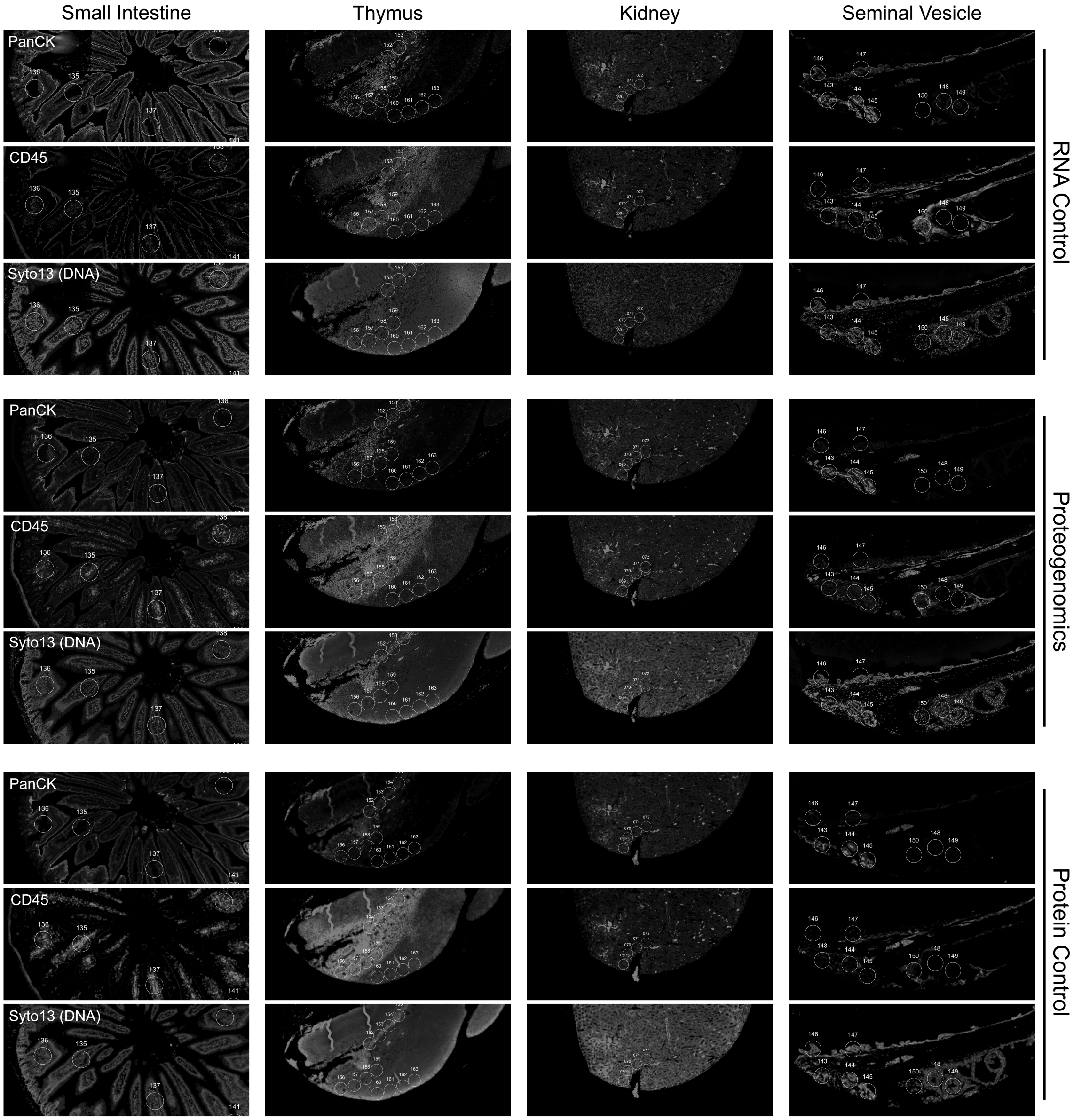

Supplementary Figure S16: Representative grey scale images of used in the assessment of spatial proteogenomic data quality across mouse tissue types. FFPE sections were stained with the GeoMx NGS Mouse Protein modules (137-plex), GeoMx Mm WTA, and antibodies against CD45 (Immune), PanCK (Tumor) and Syto13 (nuclear, DNA).

Supplementary Figure S17

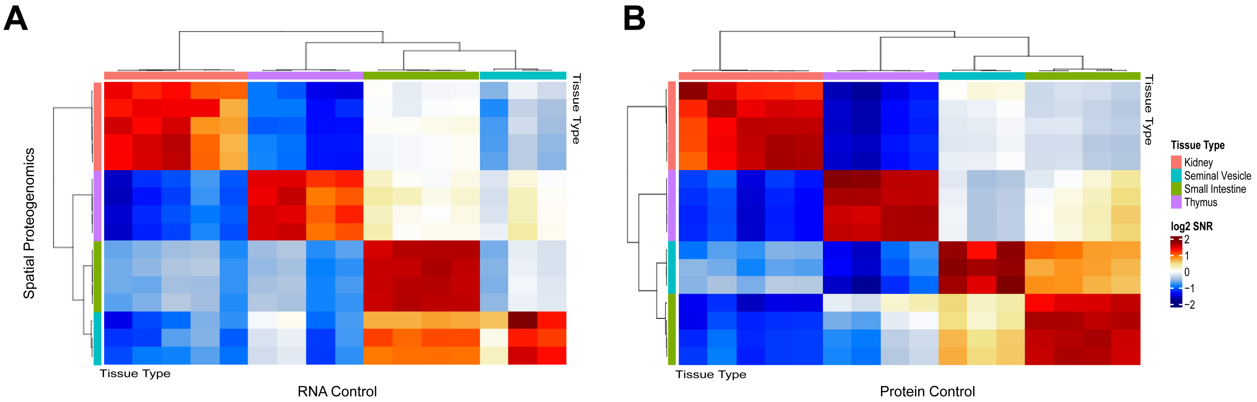

Supplementary Figure S17: ROI-to-ROI comparison of the proteogenomic data to the single analyte controls. Mouse FFPE sections were stained with 137-plex GeoMx NGS Mouse Protein modules and GeoMx Mm WTA. The Pearson's *R* was calculated between each ROI from the proteogenomic assay against all ROIs in the single analyte (**A**) RNA and (**B**) protein controls. Protein targets with SNR $\geq$ 3 and RNA targets with SNR $\geq$ 4 were used in the analysis.

Supplementary Figure S18

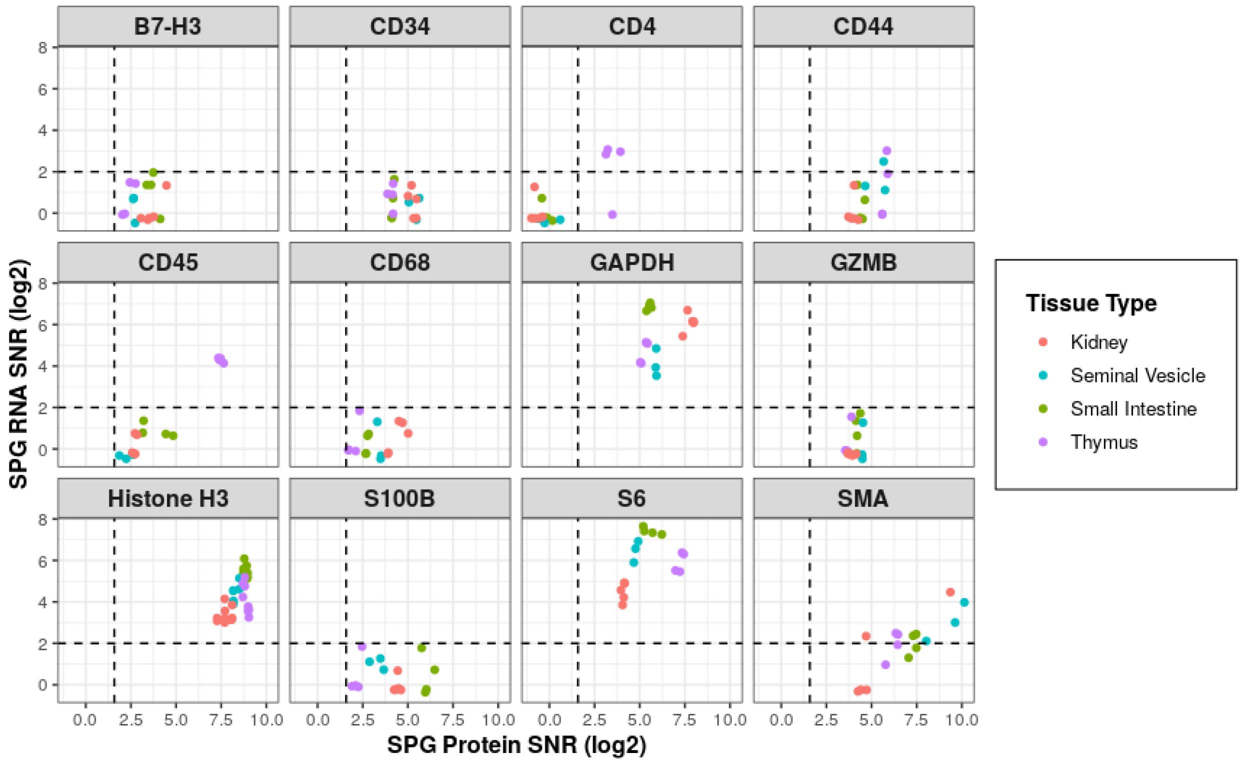

Supplementary Figure S18: Expression levels of select matching RNA and protein targets in several mouse tissue types. For protein targets with SNR ≥ 3 and the respective RNA target with SNR ≥ 4, a pairwise scatter plot was generated to visualize the concordance between respective analytes.
