# Supplementary material for: Ultra High-plex Spatial Proteogenomic Investigation of Giant Cell Glioblastoma Multiforme Immune Infiltrates Reveals Distinct Protein and RNA Expression Profiles
Source: Cancer Res Commun. 2023 May 3;3(5):763–79. doi: 10.1158/2767-9764.CRC-22-0396 (PMC10155752; doi:10.1158/2767-9764.CRC-22-0396)
Supplement: Supplementary Figures S19-S22 — Supplementary Figure S19: Representative images used in the assessment of spatial proteogenomic data quality on Glioblastoma multiforme Grade 4. (A) Human brain glioblastoma tissue array was stained with the GeoMx NGS Human Protein modules (147-plex), WTA, and antibodies against GFAP (Astrocyte/Tumor; left panel), CD45 (Immune; middle panel) and Syto13 (Nuclei; right panel). (B) ROI were segmented into GFAP+ (left panel), CD45+ (middle panel) or Syto13 (right panel). Supplementary Figure S20: Representative images used in the assessment of spatial proteogenomic data quality on gcGBM and GBM. ROIs for gcGBM (top panel) and GBM (bottom panel) were segmented into GFAP+ (Astrocyte/Tumor; left panel), CD45+ (Immune; middle panel) and Syto13 (Nuclear, DNA; right panel). Supplementary Figure S21: Combined volcano plot resulting from the differential expression analysis between GBM and gcGBM for all AOIs within the left frontal lobe for RNA (●) and protein (♦) analytes. Targets with significantly differential expression are highlighted either orange (P-value < 0.05), green (FDR < 0.05) or blue (FDR < 0.001); whereas targets in grey show no significant difference in expression. Genes with significantly differential expression are highlighted either orange (P-value <0.05), green (FDR < 0.05) or blue (FDR <0.001); whereas genes in grey show no significant difference in expression. A subset of differentially expressed genes are labeled. Supplementary Figure S22: Differential protein expression analysis between GBM and gcGBM. Volcano showing the differential protein expression profiles for (A) CD45- and (B) GFAP-enriched segments within the left frontal lobe. Proteins with significantly differential expression are highlighted either orange (P-value <0.05), green (FDR < 0.05) or blue (FDR < 0.001), whereas proteins in grey show no significant difference in expression. A subset of differentially expressed proteins are labeled. [file crc-22-0396-s05.pdf]

Supplementary Figure S19

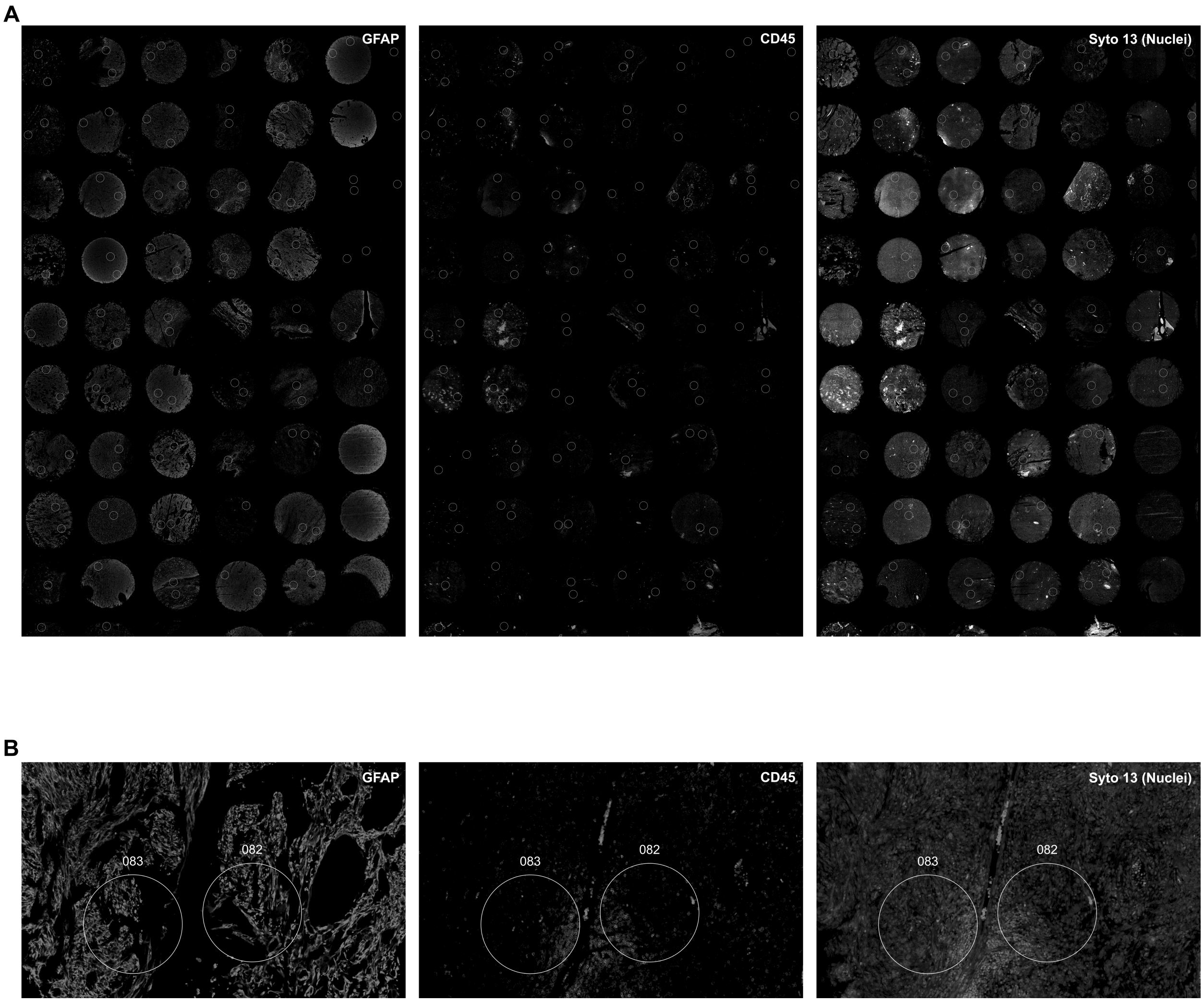

Supplementary Figure S19: Representative images used in the assessment of spatial proteogenomic data quality on Glioblastoma multiforme Grade 4. (A) Human brain glioblastoma tissue array was stained with the GeoMx NGS Human Protein modules (147-plex), WTA, and antibodies against GFAP (Astrocyte/Tumor; left panel), CD45 (Immune; middle panel) and Syto13 (Nuclei; right panel). (B) ROI were segmented into $GFAP^{+}$ (left panel), $CD45^{+}$ (middle panel) or Syto13 (right panel).

Supplementary Figure S20

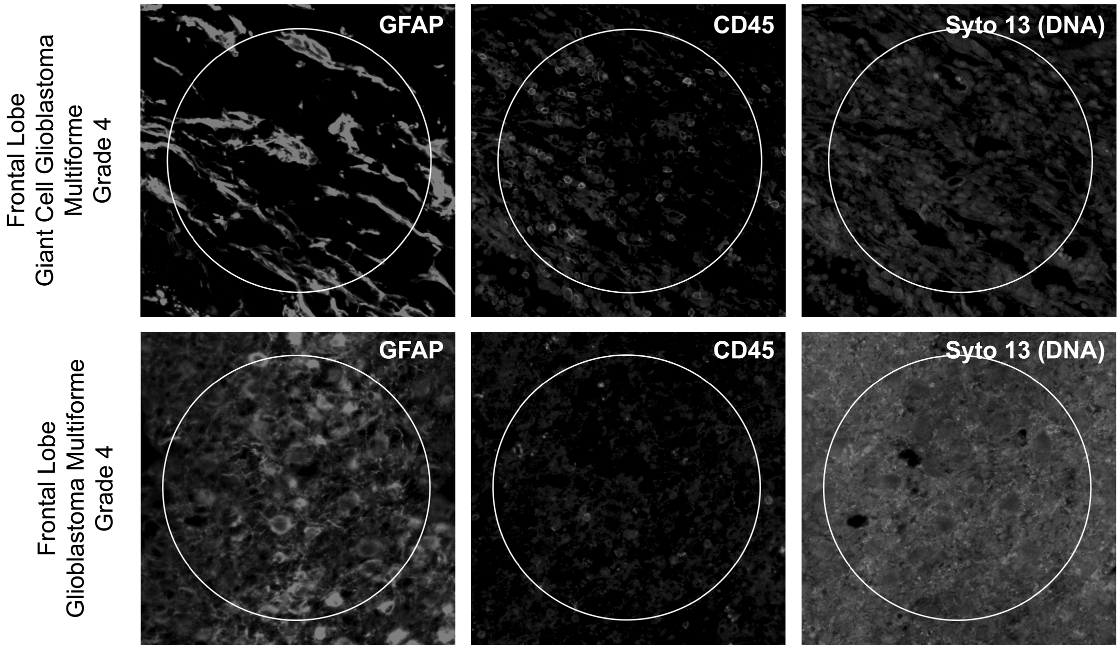

Supplementary Figure S20: Representative images used in the assessment of spatial proteogenomic data quality on gcGBM and GBM. ROIs for gcGBM (top panel) and GBM (bottom panel) were segmented into GFAP^{+} (Astrocyte/Tumor; left panel), CD45^{+} (Immune; middle panel) and Syto13 (Nuclear, DNA; right panel).

Supplementary Figure S21

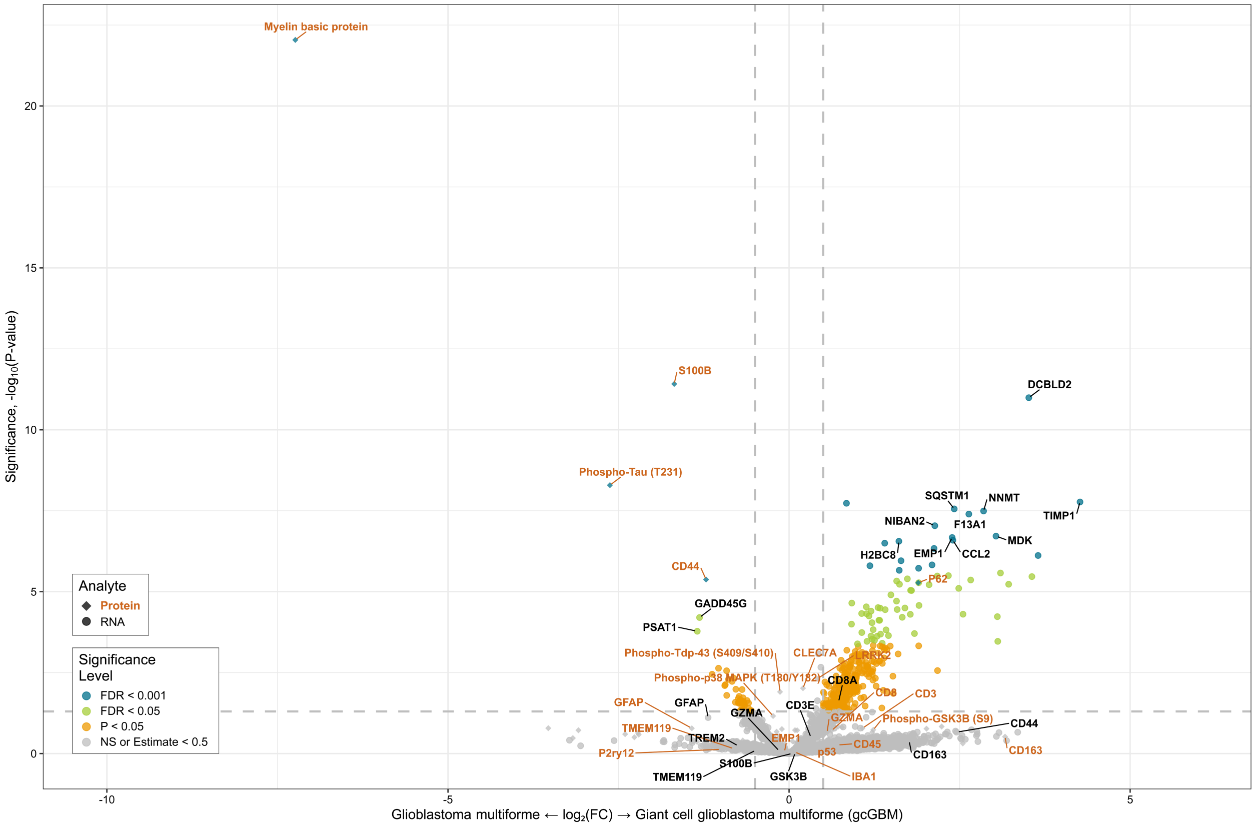

Supplementary Figure S21: Combined volcano plot resulting from the differential expression analysis between GBM and gcGBM for all AOIs within the left frontal lobe for RNA (●) and protein (♦) analytes. Targets with significantly differential expression are highlighted either orange (P-value < 0.05), green (FDR < 0.05) or blue (FDR < 0.001); whereas targets in grey show no significant difference in expression. Genes with significantly differential expression are highlighted either orange (P-value < 0.05), green (FDR < 0.05) or blue (FDR <0.001); whereas genes in grey show no significant difference in expression. A subset of differentially expressed genes are labeled.

Supplementary Figure S22

A

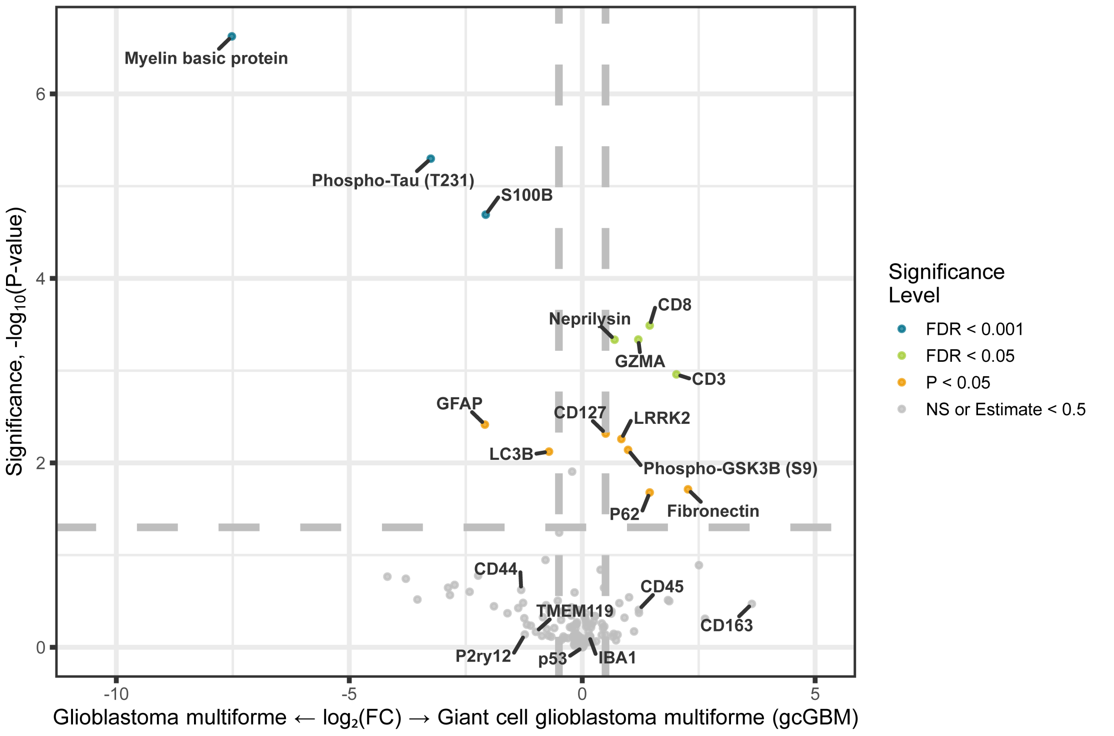

B

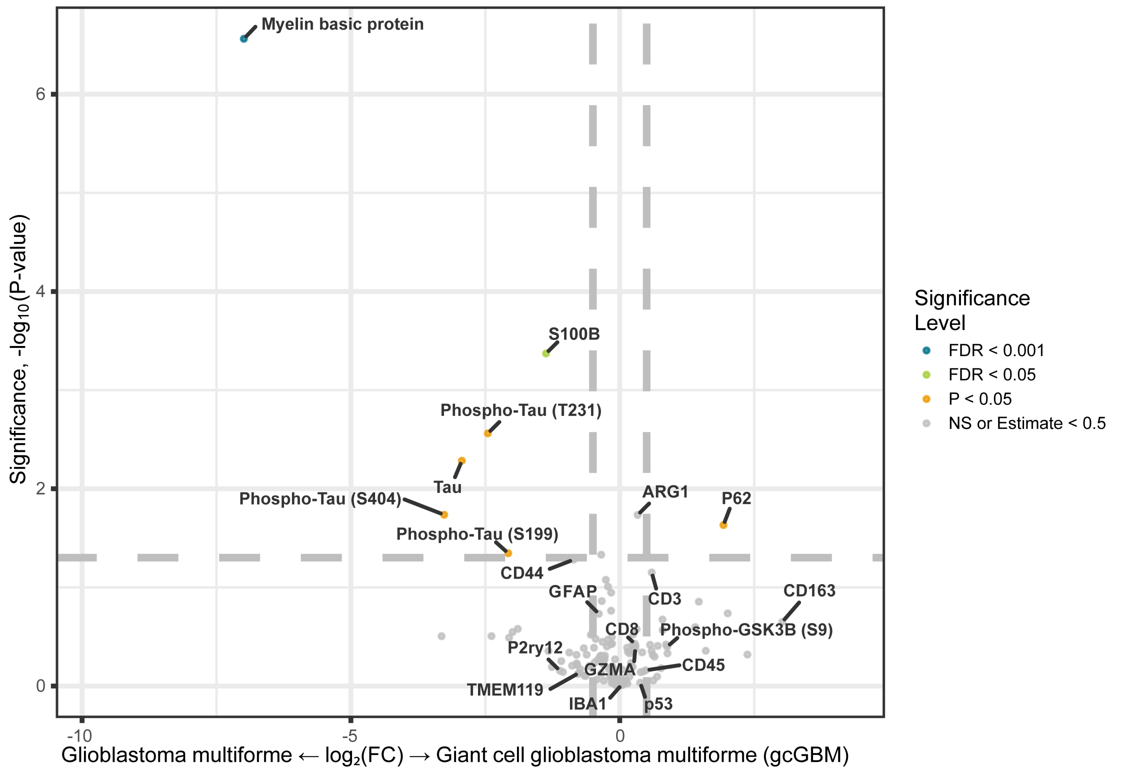

Supplementary Figure S22: Differential protein expression analysis between GBM and gcGBM. Volcano showing the differential protein expression profiles for (**A**) CD45- and (**B**) GFAP-enriched segments within the left frontal lobe. Proteins with significantly differential expression are highlighted either orange (*P*-value < 0.05), green (FDR < 0.05) or blue (FDR < 0.001), whereas proteins in grey show no significant difference in expression. A subset of differentially expressed proteins are labeled.
