# Supplementary material for: Ultra High-plex Spatial Proteogenomic Investigation of Giant Cell Glioblastoma Multiforme Immune Infiltrates Reveals Distinct Protein and RNA Expression Profiles
Source: Cancer Res Commun. 2023 May 3;3(5):763–79. doi: 10.1158/2767-9764.CRC-22-0396 (PMC10155752; doi:10.1158/2767-9764.CRC-22-0396)
Supplement: Supplementary Methods S1 — Protocol for proteogenomic workflow [file crc-22-0396-s06.pdf]

Supplementary Methods

Supplementary Methods S1

Quick Guide: Spatial Proteogenomic Assay

Please refer to the GeoMx DSP Spatial Proteogenomic Protocol Manual (MAN-10158) for detailed instructions.

Day 1:

1. Bake slides @ 60°C for 30 min to 3 hours then cool to room temp.
2. Set up the Leica BOND RX/RX^{m}.
   a. Fill reagent containers and add them to the BOND Research Detection System:
      i. **1X BOND WASH:** 30 mL into Home Buffer Open Container
      ii. **Proteinase K:** 0.1 µg/mL in 1X PBS (see Table 1); 500 µL per slide plus 500 µL dead volume in BOND Titration Container.
   b. Load the BOND Research Detection System.
   c. From Slide Setup, select **Add Study**. Enter study information as shown below:
      i. **Dispense volume:** 150 µL
      ii. **Preparation protocol:** Bake and Dewax
      iii. Select Ok
   d. Select **Add Slide**. Enter study information as shown below:
      i. **Tissue type:** Test tissue
      ii. **Dispense volume:** 150 µL
      iii. **Staining mode:** Single and Routine
      iv. **Process:** IHC
      v. **Marker:** *Negative
      vi. **Staining:** Proteogenomic slide prep for Staining
      vii. **Preparation:** *Bake and Dewax
      viii. **HIER:** Select appropriate incubation time and temp based on the tissue type (**Table 1**)
      ix. **Enzyme:** Select appropriate incubation time based on the tissue type (**Table 2**)
   e. Print and attach labels to the slides.
   f. Load slides with cover tiles into the slide tray.
   g. Load and run your slides.

Table 1: Target Retrieval times and temps by tissue type

Tissue Type	Target Retrieval
Breast	*HIER 10 min with ER2 @ 100°C
Cell pellets	*HIER 10 min with ER2 @ 85°C
Colorectal	*HIER 20 min with ER2 @ 100°C
Melanoma	*HIER 20 min with ER2 @ 100°C
Mouse tissue	*HIER 10 min with ER2 @ 100°C
NSCLC	*HIER 20 min with ER2 @ 100°C
Prostate tumor	*HIER 20 min with ER2 @ 100°C
Tonsil	*HIER 20 min with ER2 @ 100°C

Table 2: Proteinase K digest concentration and times by tissue type

Tissue Type	Proteinase K Digest
Breast	0.1 µg/mL for 15 min
Cell pellets	0.1 µg/mL for 5 min
Colorectal	0.1 µg/mL for 15 min
Melanoma	0.1 µg/mL for 15 min
Mouse tissue	0.1 µg/mL for 15 min
NSCLC	0.1 µg/mL for 15 min
Prostate tumor	0.1 µg/mL for 15 min
Tonsil	0.1 µg/mL for 15 min

3. When the run has completed, remove the cover tiles and place slides in 1X PBS.
4. In Situ Hybridization (Overnight)
    a. Clean hybridization chamber with RNase Away and prepare chamber with damp Kimwipes.
    b. Warm Buffer R to room temp before opening.
    c. Thaw RNA detection probes on ice. Before use, mix thoroughly by pipetting.
    d. Make hybridization solution following Table 3 (n = number of slides).

Table 3: Hybridization solution equation

Panel Configuration	Buffer R	RNA Probe Mix	Custom spike-in	Custom spike-in	DEPC-treated water	Final Volume
Atlas (WTA, CTA) alone	200 µL x *n*	25 µL x *n*	n/a	n/a	25 µL x *n*	250 µL x *n*
1 custom spike-in	200 µL x *n*	25 µL x *n*	12.5 µL x *n*	n/a	12.5 µL x *n*	250 µL x *n*
2 custom spike-ins	200 µL x *n*	25 µL x *n*	12.5 µL x *n*	12.5 µL x *n*	n/a	250 µL x *n*

    e. Handling one slide at a time, wipe excess PBS and place in hyb chamber.
    f. Add 200 µL hyb solution and gently apply a Hybrislip coverslip, avoiding air bubbles.
    g. Repeat for all slides.
    h. Place hyb chamber in the over to hybridize overnight at 37°C.

Day 2:

1. Perform stringent washes (1 hour)
    a. Warm 100% formamide to room temp before opening.
    b. Staining jars should be cleaned with RNase AWAY before use.
    c. Make Stringent Wash by mixing equal parts 100% formamide and 4X SSC.
    d. Fill two staining jars with Stringent Wash and preheat them to 37°C in water bath.
    e. Dip slides in 2X SSC allowing coverslip to slide off.
    f. Wash two times in Stringent Wash Solution for 25 min each at 37°C.
    g. Wash two times in 2X SSC for 5 min each at room temp.

2. Primary antibody incubation (Overnight)
    a. Wash one time in 1X TBS-T for 5 min at room temp.
    b. Handling one slide at a time, remove excess buffer, draw a closed barrier with a hydrophobic pen and place in humidity chamber.
    c. Block by applying 200 µL Buffer W to each of the slides. Incubate at room temp for 1 hr in a closed humidity chamber.
    d. Thaw Ab mix on ice and protected from light.
    e. Make working antibody solution (see Table 4).

Table 4: Reagent volumes for Working Antibody Solution

Core Ab Mix	Module 1 Ab Mix	Module 2 Ab Mix	Each addl. Module Ab Mix	Morphology Marker 1	Morphology Marker 2	Each addl. Morphology Marker	Buffer W	Total Volume
8 µL x *n*	8 µL x *n*	8 µL x *n*	8 µL x *n*	5.5 µL x *n*	5.5 µL x *n*	5.5 µL x *n*	(up to 200 µL) x *n*	200 µL x *n*

    f. Cover tissue with 200 µL working antibody solution.
    g. Incubate overnight in closed humidity chamber at 4°C protected from light.

Day 3:

1. Post-Fix (1.75 hours)
    a. Wash three times for 10 min each in 1X TBS-T.
    b. Carefully tap each slide on a fresh Kimwipe to remove excess solution.
    c. Cover tissue with 200 µL 4% PFA and incubate 30 min at room temp in a closed humidity chamber.
    d. Wash two times for 5 min each in 1X TBST.
    e. Dilute SYTO 13 1:10 using 1X TBS.
    f. Cover tissue with 200 µL of diluted SYTO 13 and incubate 15 min at room temp in closed humidity chamber.
    g. Wash slide in 1X TBS-T.
    h. Scrape off hydrophobic pen and store slides in 1X TBS-T.
2. Load slides and operate the GeoMx DSP instrument for aspirate collection according to the GeoMx-NGS DSP Instrument manual (MAN-10152).

Day 4:

1. NGS Library Preparation
    a. After the GeoMx run is complete, proceed to NGS library preparation as described in the GeoMx DSP NGS Readout User Manual (MAN-10153).
    b. Follow the steps of conventional (single analyte) GeoMx NGS Library Preparation, noting this specific instruction for the Proteogenomic assay protocol:
        i. In the first step of the NGS Library Prep, the aspirates in the collection plate are dried down and then rehydrated. **Rehydrate proteogenomic assay samples in 10 µL of DEPC-treated water.**
    c. Remaining library preparation, purification and sequence loading steps should be followed as written in the GeoMx DSP NGS Readout Manual.
    d. A sequencing depth of 650 reads per μm^2 of collection area is recommended.
